# The Head Turning Modulation System: An Active Multimodal Paradigm for Intrinsically Motivated Exploration of Unknown Environments

**DOI:** 10.3389/fnbot.2018.00060

**Published:** 2018-09-21

**Authors:** Benjamin Cohen-Lhyver, Sylvain Argentieri, Bruno Gas

**Affiliations:** CNRS, Institut des Systèmes Intelligents et de Robotique, Sorbonne Université, Paris, France

**Keywords:** multimodal perception, attention, motivation, active learning, binaural audition

## Abstract

Over the last 20 years, a significant part of the research in exploratory robotics partially switches from looking for the most efficient way of exploring an unknown environment to finding what could motivate a robot to autonomously explore it. Moreover, a growing literature focuses not only on the topological description of a space (dimensions, obstacles, usable paths, etc.) but rather on more semantic components, such as multimodal objects present in it. In the search of designing robots that behave autonomously by embedding life-long learning abilities, the inclusion of mechanisms of attention is of importance. Indeed, be it endogenous or exogenous, attention constitutes a form of intrinsic motivation for it can trigger motor command toward specific stimuli, thus leading to an exploration of the space. The Head Turning Modulation model presented in this paper is composed of two modules providing a robot with two different forms of intrinsic motivations leading to triggering head movements toward audiovisual sources appearing in unknown environments. First, the Dynamic Weighting module implements a motivation by the concept of Congruence, a concept defined as an adaptive form of semantic saliency specific for each explored environment. Then, the Multimodal Fusion and Inference module implements a motivation by the reduction of Uncertainty through a self-supervised online learning algorithm that can autonomously determine local consistencies. One of the novelty of the proposed model is to solely rely on semantic inputs (namely audio and visual labels the sources belong to), in opposition to the traditional analysis of the low-level characteristics of the perceived data. Another contribution is found in the way the exploration is exploited to actively learn the relationship between the visual and auditory modalities. Importantly, the robot—endowed with binocular vision, binaural audition and a rotating head—does not have access to prior information about the different environments it will explore. Consequently, it will have to learn in real-time what audiovisual objects are of “importance” in order to rotate its head toward them. Results presented in this paper have been obtained in simulated environments as well as with a real robot in realistic experimental conditions.

## 1. Introduction

One of the most critical and important task humans are able to do is to explore unknown environments, topologically or semantically, while being able to create internal representations of them for localization in it and interaction with it. Such cerebral representations, or maps as it is often referred to O'Keefe and Nadel (1978) and Cuperlier et al. (2007), enable humans and animals in general to gather and organize perceptual cues (visual, acoustic, tactile, olfactory, proprioceptive…) in semantic components. In parallel, in the mobile robotics community, exploration of unknown environments has been one of the most important fields studied, back to the artificial turtles of Walter (1951) and later to the vehicles of Braitenberg (1986). Indeed, being able for a mobile robot to simultaneously (i) map the world it is exploring, (ii) locate itself in it, and (iii) trigger relevant motor actions for further exploration (i.e., the three key tasks to perform in an exploration scheme according to Makarenko et al., 2002), has shown to be a hard, but critical for robots' existence, problem to solve. While many artificial systems have been implemented with the sole purpose of *exploring the most of an environment* with only efficiency as a goal (Smith et al., 1987; Henneberger et al., 1991; Montemerlo et al., 2002; Carrillo et al., 2015), some more recent algorithms emerged on the basis of the precursor works of Berlyne (1950, 1965), who stated that *Motivation* is a fundamental mechanism in spontaneous exploratory behaviors in humans. Following this principle, exploration would not be driven by a goal defined by an external agent (such as the human experimenter) but rather by internal goals defined by the robot itself, that is *intrinsic* motivations (Ryan and Deci, 2000; Oudeyer and Kaplan, 2008). Amongst them are the motivations by *Curiosity*, first mathematically modeled by Schmidhuber (1991), by *Uncertainty* (Huang and Weng, 2002), by *Information gain* (Roy et al., 2001), or by *Empowerment* (Capdepuy et al., 2007). Intrinsic motivation has extensively been used during the last 20 years in several powerful systems, in particular by Oudeyer et al. (2007) with the development of the Independent Adaptive Curiosity algorithm (IAC) and the later updated systems (R-IAC, Baranes and Oudeyer, 2009 and SAGG-RIAC, Baranes and Oudeyer, 2010). Systems based on such motivations to explore/understand an environment incorporate in particular the notion of *reward*, a principle that is of high importance in learning in primates and humans (Rushworth et al., 2011). As such, these systems are particularly suited for adaptive life-long learning robots for they bring to them wider motivations to react to their environments: instead of compelling the robot to “*explore as quickly as possible every inch of the room*”, it becomes closer to “*just be curious*”. But beyond the topological characteristics of unknown environments, their content also provides valuable information for the robots internal representation of the world (object formation, their affordance, etc.). Then, while one of the most predominant issue in driving topological exploration is to decide what is the next point or area to explore, semantical exploration can be also introduced to determine what is the next component to discover. Such considerations are close to attentional behaviors, which have also been extensively studied (Downar et al., 2000; Hopfinger et al., 2000; Corbetta and Shulman, 2002; Corbetta et al., 2008; Petersen and Posner, 2012).

Among others, saliency is known to be a key feature in attention thanks to its sensitivity to discontinuity in perceived data. A significant literature can be found on saliency-driven exploration: eye saccades modelization (Itti et al., 1998; Oliva et al., 2003; Le Meur and Liu, 2015), detection of auditory salient events (Kayser et al., 2005; Duangudom and Anderson, 2007), or audiovisual objects exploration (Ruesch et al., 2008; Tsiami et al., 2016). However, most of these models propose either a solely off-line solution requiring prior training from large databases, or an immutable saliency characterization of events. Moreover, the fact that these models only deal with the low-level characteristics of the perceived data leads often to an absence of wider context inclusion, be it through a form of memory, or through the semantics of the events. In addition, saliency can somehow differ from importance, depending on the task to accomplish: attention can be driven by behaviorally important but not salient stimuli while, on the other hand, very salient stimuli but showing no behavioral importance can be disregarded by the attentional networks (Corbetta and Shulman, 2002; Indovina and Macaluso, 2007). However, it is worth mentioning the interesting feature of the multimodal model of salience of (Ruesch et al., 2008) as the implementation of an additional inhibition map to the ones already used for saliency. Such map promotes the exploration of unknown parts of the environments and avoids deadlock situations caused by local minima. This has also to be brought close to the notion of motivations for exploration mentioned above since a form of Curiosity is here implemented.

In this paper is presented a computational system, The Head Turning Modulation system (HTM), which aims at giving a mobile robot endowed with binaural hearing, binocular vision and a rotating head, the ability to decide which audiovisual sources present in unknown environments are worth the robot's attention. The principle of attention mentioned in this paper is based on the prime definition originating from James (1890): “Everyone knows what attention is. It is the taking possession by the mind, in clear and vivid form, of one out of what seem several simultaneously possible objects or trains of thought”. More particularly, the proposed HTM system is dedicated to the implementation of an overt and endogenous (Driver and Spence, 1998; Le Meur et al., 2006) attentional reaction: *the head turning*. This reaction, known to be one of the attentional behavior involved in the mechanism of *attention reorientation* to unpredictable stimuli (Thompson and Masterton, 1978; Corbetta et al., 2008; Corneil et al., 2008), aims at bringing the visual sensors in front of the sources of interest hence enabling the robot to gather and analyze additional data. In addition, the HTM system provides the robot with an adaptive enough online learning behavior so that it can endlessly integrates new useful information to its self-created audiovisual database. However, this learning relying intensively upon the triggering of head movements, it is also necessary for the robot to understand when this knowledge is robust and relevant enough, thus not requiring further motor reaction. The HTM is part of a much wider system, implemented as the Two!Ears software^1^, which aims at providing a computational framework for modeling active exploratory listening that assigns meaning to auditory scenes. More precisely, it consists in perceiving and analyzing a multimodal world through a paradigm that combines a classical bottom-up signal-driven processing step together with a top-down cognitive feedback. In there, the HTM is in charge of building an internal semantic map of the explored environment, made of localized audiovisual objects coupled with their respective semantic importance, the so-called congruence.

In comparison with other works, the proposed system is described as a *real-time* (Huang et al., 2006) and *online* behavioral unit, which is always able to learn new situations while also taking advantage of its previous experience of the past environments. In terms of architecture, the proposed system receives data from several “experts” from the Two!Ears software, i.e., computational elements specialized in very particular tasks, such as the identification of sounds or images. It means that the HTM system is placed right after these experts, and thus receives already highly interpreted data. Two main parts constitute the system: an attentional component, the *Dynamic Weighting* module (Walther and Cohen-Lhyver, 2014), and a learning component, the *Multimodal Fusion & Inference* module (Cohen-Lhyver et al., 2015). On the one hand, the dw module is dedicated to the analysis of perceived audiovisual objects through the concept of *Congruence*, defined as a semantic saliency and rooted in the principle of optimal incongruity (Hunt, 1965). The dw module implements a form of motivation by surprise for it favors unexpected audiovisual events. On the other hand, the mfi module learns the association between auditory and visual data in order to make the notion of *multimodal object* arise from potentially erroneous data of the aforementioned experts. The mfi module implements a form of motivation by reduction of uncertainty for it aims at consolidating as much as needed its knowledge about the audiovisual objects that the robot encounters. This learning serves two purposes. First, it might improve the robustness and reliability of the classification (Droniou et al., 2015). Secondly, it allows the system to perform missing information inference (Bauer and Wermter, 2013), as when an object is placed behind the robot thus having only access to the auditory information.

The paper is organized as follows. To begin with, the overall Two!Ears framework, together with the notations used all along the paper, are introduced in a first section. On this basis, the overall HTM system is thoroughly presented in a second section: after a short insight into the HTM system architecture, the way the dw module and the mfi module operate is formalized. This section also presents their respective evaluation in simulated conditions. Then, the combination of the two modules is investigated and the evaluation of the approach in real experimental conditions, that is including a real robot in a real environment, is made. Finally, a conclusion ends the paper.

## 2. Context and notations

This section presents the context in which is rooted the proposed HTM system. All the forthcoming development has taken place inside a specific computational architecture aiming at modeling an integral, multimodal, intelligent and active auditory perception and experience. This model physically uses two human-like ears and visual inputs to make a mobile robot able to interactively explore unknown environments, see Two!Ears (2016b). Among other applications, this modular architecture targets evaluation of bottom-up audiovisual processing coupled with top-down cognitive processes. The proposed HTM system relies also on this top-down and bottom-up paradigm providing the robot with a reliable internal representation of its audiovisual environment. To begin with, a short overview of the overall architecture is proposed in a first subsection. A second subsection introduces the notations used all along the paper, together with all the notions required to understand the HTM system.

### 2.1. Global framework

**Figure 1:**
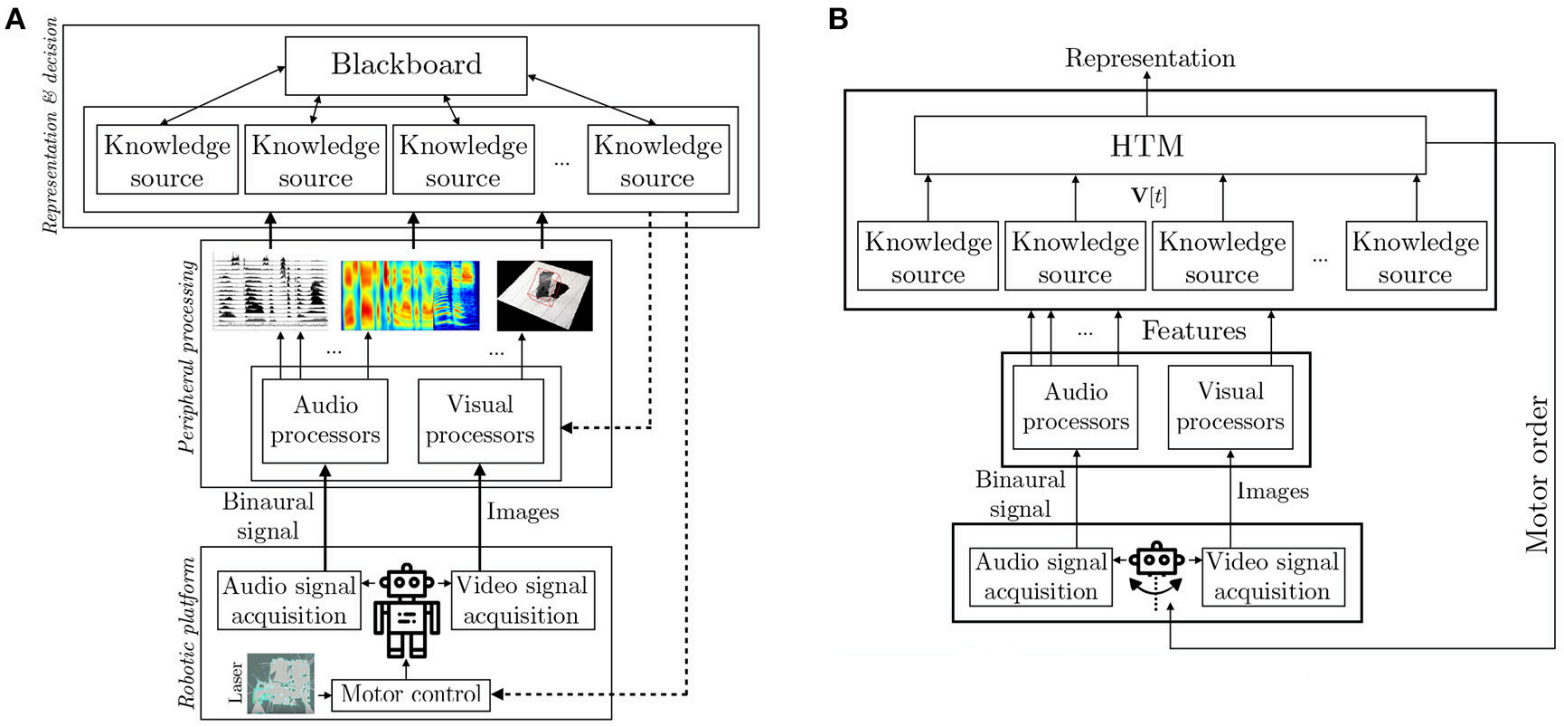
Two!Ears architecture. **(A)** On the basis on audio and visual data, features are extracted to provide a compact description of the data. Several audio and visual experts (or Knowledge Sources, KS) exploit these features to analyze the signals. Each KS is specialized in one task: recognition of one type of sound, localization, separation, etc. All experts share their knowledge through a blackboard system, thus producing an internal representation of the world. On this basis, the overall system (but also individual KSs) can decide to modulate either the feature extraction step, or the action of the robot. The proposed HTM system, implemented as a KS, is–among others–responsible for this last modulation. **(B)** Focus on the implementation of the HTM system inside this architecture.

All the forthcoming developments have been conducted inside the multilayer Two!Ears architecture, see Figure 1A. This figure highlights two different pathways: first, a classical bottom-up processing way, where raw data coming from the sensors (microphones and cameras) are first analyzed (features extraction step), processed (through some specialized pattern recognition algorithms) and interpreted (representation and decisional layers). All of the above is computed by dedicated Knowledge Sources (KS). The main contribution of this architecture is that all these layers are highly and dynamically parameterizable: for instance, most of the feature extractions parameters (for audio data, one could cite the number of Gammatone filters used, their repartition on the frequency scale, etc.) can be changed on the fly. In general, the decision to change parameters comes from upper layers, resulting in a top-down pathway, also involving decisions concerning the movement of the robot itself. Such decisions concerning the robot actions are of particular importance, especially when dealing with attention reorientation and scene understanding for they add adaptability to new and unpredictable events.

The HTM system inside the Two!Ears architecture shown in Figure 1B is implemented as a Knowledge Source (KS). It gets data from other KSs available in the architecture through a blackboard (Schymura et al., 2014) (which can be seen, with a rough simplification, as a data structure), and provides as an output a proposition for a motor command, together with an interpreted representation of the robot's world. One originality of the approach is that the HTM system is placed behind other KS, thus not working directly with the features extracted from the raw audio and visual signals. All of the KSs the HTM relies upon contribute to the scene analysis and are fused by the HTM into a representation of the world that spans wider in time than the one provided by the individual KSs. This representation is made of all the unknown environments explored by the robot, each of them being characterized by the audiovisual objects observed there in an allocentric representation, coupled with an additional semantic layer formalized through the notion of Congruence. The data used by the HTM, together with their notations are described in the following section.

### 2.2. Definitions and notations

The HTM system only relies upon KSs outputs to analyze the unknown environments the robot explores. These KSs are classification experts specialized in the recognition of audio or visual frames (Two!Ears, 2016a), classified in terms of audio classes $c_{i}^{a}$, with *i* = 1, …, *N*_*a*_ (such as $c_{i}^{a}\in \left\{\text{voice},\text{barking},\text{yelling},\ldots \right\}$) or visual classes $c_{k}^{v}$, with *k* = 1, …, *N*_*v*_ (such as $c_{k}^{v}\in \left\{\text{DOG},\text{BABY},\text{MALE PERSON},\ldots \right\}$) with *N*_*a*_ and *N*_*v*_ the number of audio and visual classes, respectively. All classifiers are mutually independent, each providing a probability $p_{i}^{a}\left[t\right]$ and $p_{k}^{v}\left[t\right]$ for the frame at time *t* to belong to the class they represent. All these probabilities are regrouped by modality in the two vectors ${\text{P}}^{a}\left[t\right]=\left(p_{1}^{a}\left[t\right],\ldots ,p_{N_{a}}^{a}\left[t\right]\right)$ and ${\text{P}}^{v}\left[t\right]=\left(p_{1}^{v}\left[t\right],\ldots ,p_{N_{v}}^{v}\left[t\right]\right)$. In addition, the Two!Ears architecture provides *N*_θ_ localization experts (May et al., 2011; Ma et al., 2015), aiming at localizing audio and/or visual events in the horizontal plane with respect to the robot. Each of them outputs a probability $p_{\theta_{u}}^{a}\left[t\right]$ and $p_{\theta_{u}}^{v}\left[t\right]$, with *u* = 1, …, *N*_θ_, for an audio and/or visual event to originate from the azimuth $\theta_{u}^{a}$ or $\theta_{u}^{v}$ (by convention, θ = 0° corresponds to an event placed in front of the robot). All these probabilities are gathered into the audio and visual localization vectors $\Theta^{a}\left[t\right]=\left(p_{\theta_{1}}^{a}\left[t\right],\ldots ,p_{\theta_{N_{\theta }}}^{a}\left[t\right]\right)$ and $\Theta^{v}\left[t\right]=\left(p_{\theta_{1}}^{v}\left[t\right],\ldots ,p_{\theta_{N_{\theta }}}^{v}\left[t\right]\right)$. In practice, all these classifiers outputs are regrouped into a single vector **V**[*t*] constituting the sole HTM system input, with

(1) $$\begin{array}{r} \begin{array}{c} \text{V}\left[t\right]=\left(\text{P}\left[t\right],\Theta \left[t\right]\right),\\ \text{with }\text{P}\left[t\right]=\left({\text{P}}^{a}\left[t\right],{\text{P}}^{v}\left[t\right]\right)\text{and}\Theta \left[t\right]=\left(\Theta^{a}\left[t\right],\Theta^{v}\left[t\right]\right). \end{array} \end{array}$$

**Figure 2:**
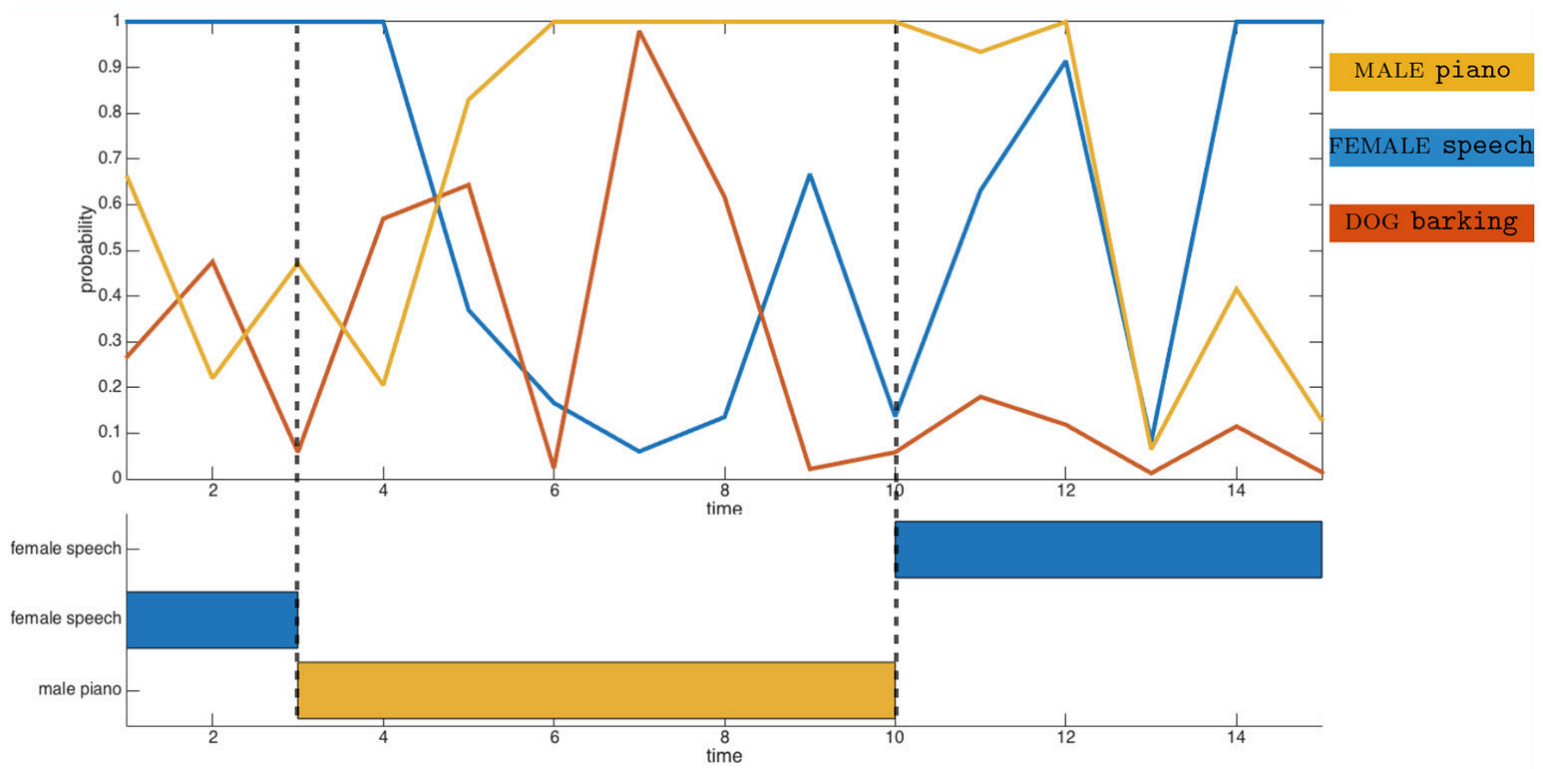
Illustration of the audio classification experts on real perceived data. **(Top)** Probabilities of the frames to belong to the corresponding audio classes. **(Bottom)** Time description of the audiovisual objects appearance.

From **V**[*t*], the HTM model attempts to build a stable and reliable internal representation of the world, environment by environment. Such a representation is obtained by transforming an audio and/or visual event Ψ_*j*_ objectively present in the environment at azimuth θ(Ψ_*j*_) and belonging to the ground truth audiovisual class $c\left({\text{Ψ}}_{j}\right)=\left\{c^{a}\left({\text{Ψ}}_{j}\right),c^{v}\left({\text{Ψ}}_{j}\right)\right\}$, into an object *o*_*j*_ perceived by the robot, i.e.,

(2) $$\begin{array}{l} \Psi_{j}=\{\theta (\Psi_{j}),c(\Psi_{j})\}\to o_{j}=\{\hat{\theta }(o_{j}),\hat{c}(o_{j})\},\\ \text{with }\hat{\theta }(o_{j})=\left\{ \begin{array}{l} \theta_{u}^{a},\text{ with }u=\arg\max_{i}(p_{\theta_{i}}^{a}),\text{ if }\theta_{u}^{a}\geq \left|\theta_{\text{HTM}}-\theta_{\text{FOV}}\right|\\ \theta_{u}^{v},\text{ with }u=\arg\max_{k}(p_{\theta_{k}}^{v})\text{ otherwise} \end{array}\right.\\ \text{ and }\hat{c}(o_{j})=\{{\hat{c}}^{a}(o_{j}),{\hat{c}}^{v}(o_{j})\}, \end{array}$$

where θ_htm_ and θ_fov_ represent the current azimuthal head position and the field of view of the camera, respectively. Then, an object *o*_*j*_ is defined by its estimated angular position $\hat{\theta }\left(o_{j}\right)$ and its estimated audiovisual class $\hat{c}\left(o_{j}\right)$ made of the estimated audio class ${\hat{c}}^{a}\left(o_{j}\right)$ and estimated visual class ${\hat{c}}^{v}\left(o_{j}\right)$. Equation (2) also indicates that the estimated angular position is obtained from the audio localization experts when the objects are out of the robot sight; otherwise, visual localization experts are exploited. Because of localization and/or classification errors, the object *o*_*j*_ might differ from the corresponding Ψ_*j*_. As an example, Figure 2 plots as a function of temporal frames experimental data from three audio classifiers outputs corresponding to the audio classes piano, speech and barking. This figure shows first that potential classification errors can obviously occur: at time *t* = 7, the barking output probability reaches about 98% while a piano sound is perceived by the robot. Additionally, the data show the temporal dynamic audio experts can exhibit: while the piano starts playing at time *t* = 3, the corresponding audio expert becomes dominant a few frames later only. This delay observed experimentally will justify later technical implementation specifics.

At this point, the notion of object already constitutes more than just a structure of data. In particular, the objects created by the HTM system embed a short-term temporal smoothing of the data **P**(*o*_*j*_) they are associated with, as

(3) $$\begin{array}{r} \text{P}\left(o_{j}\right)\left[t\right]=\frac{1}{N_{t}}\overset{n=t}{\underset{n=t-N_{t}}{\sum }}\text{P}\left(o_{j}\right)\left[n\right], \end{array}$$

where *N*_*t*_ ≤ 10 is the number of frames during which data **P** have been associated to *o*_*j*_. This temporal smoothing enables the robot to take into account its past experience of the audiovisual data the robot perceived and that have been associated with this object, but also to lower the impact of the early potential erroneous outputs from the classification experts. Indeed, experiments have shown that most of them are prone to making more errors during the very first frames of perceived events. Thus, it is one of the goal of the HTM system to make the object identical to the event, even in the presence of classification errors. In all the following, the internal representation *e*^(*l*)^ of the l-th environment being explored by the system is defined as the collection of the *N*^(*l*)^ objects inside, i.e., $e^{\left(l\right)}=\left\{o_{1},\ldots ,o_{N^{\left(l\right)}}\right\}$. Note that *N*^(*l*)^ evolves along time all along the agent life on the basis of the perceived data. Importantly, this definition of an environment—which will be augmented later on in section 3.2.1)—aims at making the difference between the topological and the semantic definition of an environment (see section 1). While the robot, through its navigation system, gets to know when a new topological environment is being explored, the HTM analyzes its audiovisual content in order to assess whether this environment is really new or if it similar to a previously explored one (as explained in section 3.2.1). In that case, this audiovisual similarity enables the robot to apply previously self-created behavioral rules making its reaction abilities way quicker. Then, one can define audiovisual categories $C^{\left(l\right)}\left(c_{i}^{a},c_{k}^{v}\right)$ of this l-th representation with

(4) $$\begin{array}{r} C^{\left(l\right)}\left(c_{i}^{a},c_{k}^{v}\right)=\left\{o_{j}\in e^{\left(l\right)},{\hat{c}}^{a}\left(o_{j}\right)=c_{i}^{a}\text{ and }{\hat{c}}^{v}\left(o_{j}\right)=c_{k}^{v}\right\}. \end{array}$$

Once the events have been interpreted as objects within the internal representation *e*^(*l*)^ of the robot, the HTM system analyses them through the notion of *Congruence*, described in the next section.

## 3. The head turning modulation system

The *Head Turning Modulation* system is an attempt to provide a binaural and binocular humanoid robot with the ability to learn by its own how to react to unpredictable events and to consequently trigger or inhibit head movements toward them. Moreover, the system is endowed with a module that provides a multimodal internal representation of the world through a real-time learning paradigm that has no access to any prior knowledge about the environments to be explored. This system, partially introduced by the authors in Cohen-Lhyver et al. (2015), Cohen-Lhyver et al. (2016), and Cohen-Lhyver (2017) is defined as a model of attention supported by an object-based representation of the world. This section will thus present separately the two constitutive modules of the HTM system. An evaluation of each of them will be presented in simulated conditions, while the evaluation of the whole system, made in real conditions, will be presented in section 4.

### 3.1. Architecture of the proposed system

**Figure 3:**
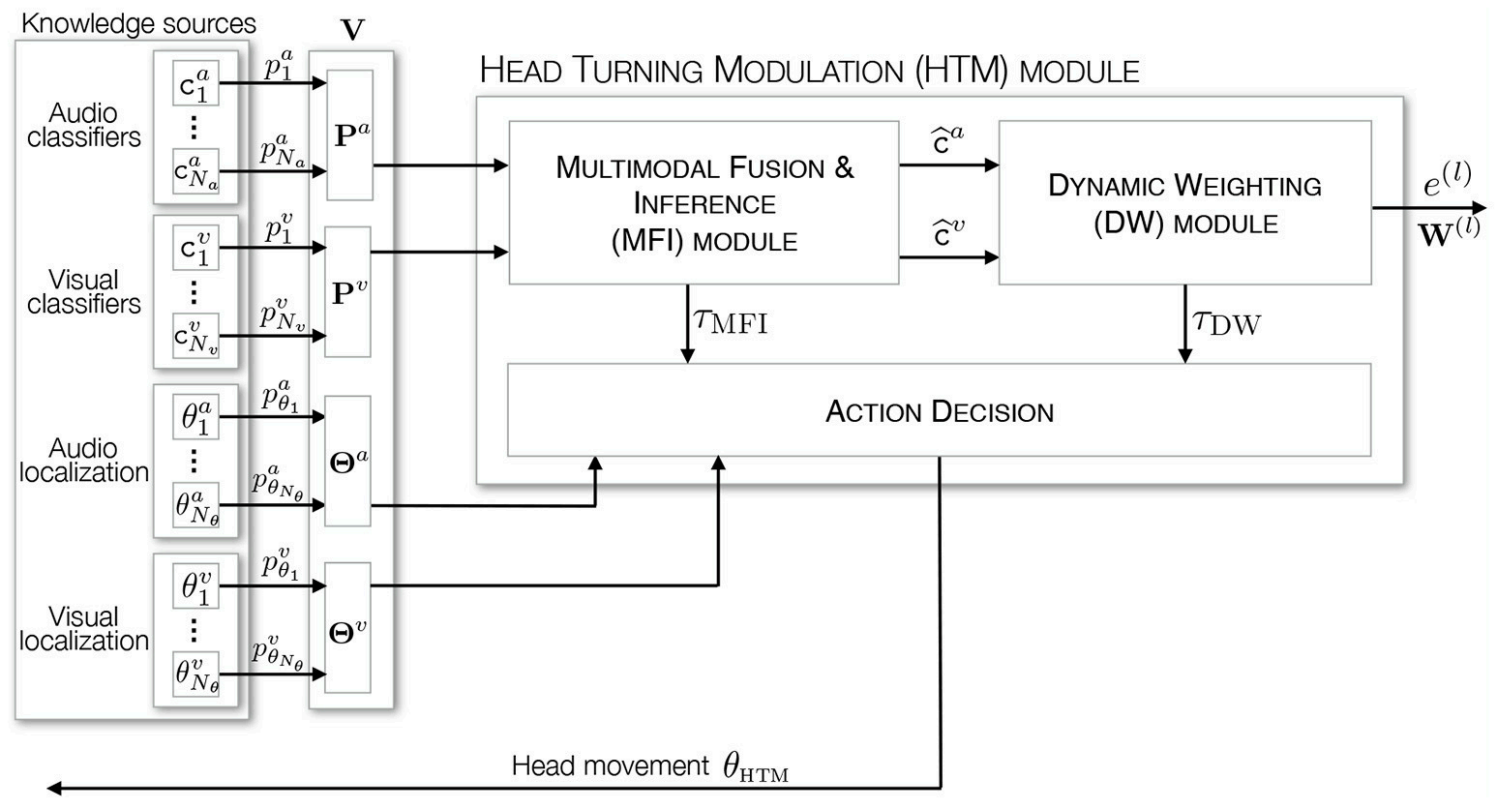
Architecture of the HTM system. It is made of two modules, dedicated to the estimation of the audiovisual class of an event and to the computation of its importance. An additional element is in charge with the respective motor orders integration and decision.

The overall architecture of the HTM system is depicted in Figure 3. It exhibits two modules inside, each of them being dedicated to one specific task. As outlined in section 2.2, the HTM inputs are made of audio and visual classifiers outputs, which are used by the first module—the Multimodal Fusion and Inference (MFI) module—to provide an estimation of the audiovisual class $\hat{c}\left(o_{j}\right)=\left\{{\hat{c}}^{a}\left(o_{j}\right),{\hat{c}}^{v}\left(o_{j}\right)\right\}$ of the currently analyzed frame. As will be shown later, such an estimation is made possible by a top-town motor feedback that allows the system to gather additional audio and visual data. On the basis on the frame estimated classification, a second module—the Dynamic Weighting (DW) module—is in charge of deciding if the currently emitting object is of interest through the computation of its congruence to the current environment. As a result, this module also exploits the motor feedback to modulate the robot attention. Since both modules require motor actions for their operations, a supplemental element is in charge with prioritizing them, depending on their respective motor activities τ_dw_ and τ_mfi_, see Figure 3. Motor decisions are taken by using the localization experts providing an estimated angle of the processed event. Finally, the overall HTM system outputs a list of interpreted objects, i.e., an internal representation of the explored environment, which can be used by other KS in the Two!Ears architecture for other tasks (modulating the exploration depending on the objects in the environment, deciding which object is of particular interest in the current scenario on the basis on the dw module module conclusions, exploiting the top-down architecture to refine the peripheral processing steps, etc.). All of these modules are introduced in the next subsections together with some intermediate illustrations and evaluations of their functioning.

### 3.2. The dynamic weighting module

The *Dynamic Weighting* module (dw module) is the attentional part of the HTM system aiming at giving the robot an hypothesis about a possible relevant audiovisual object that would present an interest to it, in the scope of the exploration of unknown environments. As already stated, this interest is formalized through the new notion of *Congruence*, thereafter detailed.

#### 3.2.1. Congruence: definition and formalization

Congruence is a notion that defines the relationship between an audiovisual event to the environment it is occurring in. It has to be brought next to the well-known and studied notion of Saliency (Treisman and Gelade, 1980; Nothdurft, 2006; Duangudom and Anderson, 2007) that describes how the perceived characteristics of a stimulus exhibit continuity, or not, with its direct surrounding. Whereas saliency is based on low-level characteristics of the signals (such as intensity, frequencies, pitch, color, contrast, etc.), Congruence relies on a higher representation of the audio and visual signals, namely the audiovisual class they belong to (see section 2.2). Congruence is thus defined as a *semantic saliency* for it relies on an already interpreted representation of the perceived data. Since the robot does not have any prior knowledge about the possible likelihood of an audiovisual event to occur in an environment, the dw module will only base its analysis on a posteriori probabilities, that is computing statistics only on what has been observed so far by the system, environment by environment. This probability of an object *o*_*j*_ to belong to a certain audiovisual category $C^{\left(l\right)}\left(c_{i}^{a},c_{k}^{v}\right)$ is thus defined as

(5) $$\begin{array}{r} p\left(o_{j}\in C^{\left(l\right)}\left(c_{i}^{a},c_{k}^{v}\right)\right)=p\left(C^{\left(l\right)}\left(c_{i}^{a},c_{k}^{v}\right)\right)=\frac{\left|C^{\left(l\right)}\left(c_{i}^{a},c_{k}^{v}\right)\right|}{N^{\left(l\right)}}, \end{array}$$

where $\left|C^{\left(l\right)}\left(c_{i}^{a},c_{k}^{v}\right)\right|$ depicts the number of objects that have already been associated to the audiovisual category $C^{\left(l\right)}\left(c_{i}^{a},c_{k}^{v}\right)$ (as a reminder, *N*^(*l*)^ corresponds to the number of objects detected so far in the l-th environment). Still following the fact that no prior knowledge is available for the robot, the system will compare this a posteriori probability to a threshold $K^{\left(l\right)}=1/N_{C^{\left(l\right)}}$ defined as the equiprobability of an object to belong to any of the categories detected so far, where $N_{C^{\left(l\right)}}$ is the number of different audiovisual categories detected in the l-th environment. Such criterion has been chosen so that minimal bias is introduced in order not to promote any audiovisual category. The criterion *K*^(*l*)^ evolves through time: the more audiovisual classes observed, the lower the criterion. Finally, the congruence decision follows:

(6) $$\begin{array}{r} o_{j}\in C^{\left(l\right)}\left(c_{i}^{a},c_{k}^{v}\right)\;\text{is incongruent}\Leftrightarrow p\left(C^{\left(l\right)}\left(c_{i}^{a},c_{k}^{v}\right)\right)\leq K^{\left(l\right)}. \end{array}$$

All the “status of congruence”, that is whether they are congruent or not, of the audiovisual categories detected by the system in a given environment are then gathered into a binary vector ${\text{W}}^{\left(l\right)}=\left\{p\left(C^{\left(l\right)}\left(c_{i}^{a},c_{k}^{v}\right)\right)\leq_{0,1}K^{\left(l\right)}\right\},\forall \left(i,k\right)$, with ≤_0,1_ a binary comparison operator. This vector of size $\left|C^{\left(l\right)}\right|$ completes the definition of environments as they become collections of objects *e*^(*l*)^ coupled with their congruence status **W**^(*l*)^. In consequence, an audiovisual class can be incongruent in an environment, but congruent in another. Since the robot would explore unknown environments during its whole life, the knowledge gained from previous explorations has to be reusable for it might speed up the exploration of new ones. Following a rule of strict inclusion of the sets of categories observed in every environment explored so far by the robot, if the set of categories detected during the exploration of an environment *e*^(*i*)^ has already been observed in a previous environment *e*^(*j*)^, then **W**^(*i*)^ = **W**^(*j*)^. This redefinition of an environment implies that there is one instantiation of the dw module per environment. In addition, even in the case where there has been a reuse of information, the rules of Congruence are still computed as if the current environment was a completely new one. Consequently, if *e*^(*j*)^ gets to differ at a point in time from *e*^(*i*)^ and that there is no other correspondence with other environments, the **W**^(*j*)^ vector computed in parallel from the beginning of the exploration of *e*^(*j*)^ will be from now on applied.

#### 3.2.2. Motor orders

Based on the congruence of all the objects, an active behavior is defined: if an object *o*_*j*_ is incongruent according to Equation (6), then it is worth focusing on it. A head movement can consequently be triggered in the direction of this object. At the opposite, if $p\left(C^{\left(l\right)}\left(c_{i}^{a},c_{k}^{v}\right)\right)>K^{\left(l\right)}$ the robot would inhibit this movement. But such a binary motor decision has several drawbacks, as demonstrated in Cohen-Lhyver et al. (2015). Among others, it presents a high sensitivity to classification errors, leading to erroneous motor decisions. Introducing a temporal weighting *w*_*o*_*j*__ of each object *o*_*j*_, inspired by the temporal dynamic of the Mismatch Negativity phenomenon (Näätänen et al., 1978), filters out efficiently most of these errors. These weights are computed thanks to two different functions, depending upon the probability $p\left(C^{\left(l\right)}\left(c_{i}^{a},c_{k}^{v}\right)\right)$, along

(7) $$w_{o_{j}}[n]=\{\begin{array}{ll} f_{\omega }^{•}[n]=1/(1+100\;e^{-2n}) & \text{if }p\left(C^{\left(l\right)}({\text{c}}_{i}^{a},{\text{c}}_{k}^{v})\right)\leq K^{\left(l\right)},\\ f_{\omega }^{{}^{\circ}}[n]=(1/1+0.01\;e^{2n})-1 & \text{else}, \end{array}$$

where $f_{\omega }^{∙}\left[n\right]$ and $f_{\omega }^{{}^{\circ}}\left[n\right]$ are increasing positive and decreasing negative functions dedicated to the weighting of incongruent and congruent objects, respectively, and *n* a time index. Note that *n* is systematically reset to 0 whenever the congruence status of the object *o*_*j*_ switches. From these weights, it is possible to decide which object has to be focused on. Such a decision is implemented through an adaptation of the GPR model (Gurney et al., 2001a,b) of the basal ganglia-thalamus-cortex loop involved in the motor order decision in humans. According to this model, all possible motor actions are expressed as channels of information which are by default inhibited by several afferent external connections. Depending on the goal or on the perceived stimuli, one of the channels is excited, thus promoting the motor action it is representing. Inspired by this functioning, all the objects perceived by the robot are similarly represented as information channels having a dedicated activity τ_dw_(*o*_*j*_). The vector of canal activities **τ**_dw_ can be then defined as

(8) $$\begin{array}{r} \tau_{\text{DW}}=\left(\tau_{\text{DW}}\left(o_{1}\right),\ldots ,\tau_{\text{DW}}\left(o_{N_{l}}\right)\right),\;\text{with }\tau_{\text{DW}}\left(o_{j}\right)=-\frac{p\left(C^{\left(l\right)}\left(c_{i}^{a},c_{k}^{v}\right)\right)}{K^{\left(l\right)}}. \end{array}$$

Thus, the higher the weight *w*_*o*_*j*__ of an object, the lowest the activity of its corresponding canal. The angle $\hat{\theta }\left(o_{j}\right)$ estimated by the audio localization expert corresponding to the canal with the lowest activity will then be selected as the winning motor order θ_dw_, i.e.,

(9) $$\begin{array}{r} \theta_{\text{DW}}=\hat{\theta }\left(o_{j}\right),\;\text{with}j=\arg\underset{l}{\min}\left(\tau_{\text{DW}}\left(o_{l}\right)\right). \end{array}$$

If two different objects *o*_*j*_ and *o*_*l*_ have the same weight *w*_*o*_*j*__ = *w*_*o*_*l*__, then their corresponding channels τ_dw_(*o*_*j*_) and τ_dw_(*o*_*l*_) have the same value. In such a case, the most recent object in the representation is promoted, thus introducing a motivation by novelty (Huang and Weng, 2002, 2004) (see also Walther et al., 2005). Then, Equation (8) is slightly modified by introducing a weight which is minimized for recently appeared objects, i.e.,

(10) $$\begin{array}{r} \tau_{\text{DW}}\left(o_{j}\right)=-\frac{p\left(C^{\left(l\right)}\left(c_{i}^{a},c_{k}^{v}\right)\right)}{K^{\left(l\right)}}\times \frac{1}{\Delta_{t}\left(o_{j}\right)},\;\text{with }\Delta_{t}\left(o_{j}\right)=t-t_{\text{emit}}\left(o_{j}\right), \end{array}$$

where Δ_*t*_(*o*_*j*_) represents the time elapsed between the object appearance *t*_emit_(*o*_*j*_) (reset to *t* when the object starts emitting again after having stopped previously) and current frame *t*. Note that the temporal smoothing introduced by Equation (3) does not influence the global reactivity to unexpected events, for the dynamics of the smoothing has the same order of magnitude to the dynamic of the weighting function in Equation (7).

#### 3.2.3. Simulations and evaluation of the DW module

The dw module aims at controlling the head movements of an exploratory robot through the notion of Congruence of perceived audiovisual objects. Thus, what is expected from the dw module is to either trigger movements toward important audiovisual sources, and to also be able to inhibit them when necessary. To illustrate this, simulations have been conducted on the basis of the Two!Ears architecture. Importantly, twelve audio classifiers and ten visual classifiers are actually implemented inside the software (Two!Ears, 2016a), making evaluation scenarios quite limited. Thus, instead of simulating raw (audio and visual) data used by real classifiers, their outputs $p_{i}^{a}\left[t\right]$ and $p_{k}^{v}\left[t\right]$ are rather simulated. Nevertheless, real conditions will be used later to evaluate the overall HTM system in section 4. Note that the forthcoming simulated localization experts are designed to provide the exact object audio and visual localization, the focus being put here on the congruence analysis performed by the dw module.

##### 3.2.3.1. Simulations

Multiple evaluation scenarios are proposed, each of them being described by the number $n_{S}$ of different sources in the simulated environment, the description of their azimuthal localization, their temporal appearance and disappearance, and their ground truth audiovisual classes *c*(Ψ)–obviously, the HTM system does not have access to any of these. The scenarios are also defined by the maximal number of simultaneously emitting sound sources $n_{sim}^{max}$. While this number never exceeds five in real extreme experimental conditions, the simulations allow to incorporate up to ten audiovisual sources. At every time step *t* of a simulation, a vector **P**[*t*] = (**P**^*a*^[*t*], **P**^*v*^[*t*]), from Equation (1) is sent to the HTM system. In the scope of the sole dw module evaluation, the estimated audio and visual classes of an event is directly obtained from **P**[*t*], i.e., on the KS outputs, according to a maximum a posteriori (MAP) estimation, with

(11) $$\begin{array}{r} {\hat{c}}_{\text{MAP}}^{a}=c_{i}^{a},\;i=\arg\underset{l}{\max}\left(p_{l}^{a}\right)\text{ and }{\hat{c}}_{\text{MAP}}^{v}=c_{k}^{v},\;k=\arg\underset{l}{\max}\left(p_{l}^{v}\right). \end{array}$$

Note that this audiovisual class estimation will be later provided by the mfi module introduced in section 3.3, as shown in Figure 3. However, because of the inevitable presence of classification errors, the corresponding audio and/or visual classes can be wrong (see Figure 2). It has been simulated through the implementation of an error rate $\varepsilon_{P}\in \left[0,100\right]\%$. At time *t*, a ground truth probability vector corresponding to the simulated event is generated. With respect to $\varepsilon_{P}$, a “wrong” classification expert index is randomly selected by drawing its value from a uniform pseudorandom number generator. Then, its associated probability is set to be the maximal value of the whole vectors **P**[*t*]. In the end, this will allow to judge the robustness of the approach to such classification errors.

Like proposed in Girard et al. (2002), the performance of the system is partially evaluated in comparison with a virtual “naive robot” noted ℜ_*n*_. In particular, ℜ_*n*_ will systematically turn its head toward any audiovisual source occurring in the environment, independently of its importance. For now, the simulations are made with an important restriction (explained and justified later): all the sources are in the field of view of the robot, i.e., the robot always has access to visual data.

##### 3.2.3.2. Evaluation 1: head movements modulation by the dw module

A rather complex environment is used in the following to illustrate the functioning of the dw module: $n_{S}=10$ audiovisual sources are present with a maximum of $n_{sim}^{max}=7$ simultaneously emitting sources. At first, let's focus on the ability of the dw module to modulate head movements by selecting only the sources of importance through the congruence analysis. Here will only be assessed the behavioral role conferred by the dw module to the robot; in consequence the simulated classification experts will be set as outputting perfect data, that is $\varepsilon_{P}=0$ (evaluations with higher error rates are made later in the paper).

**Figure 4:**
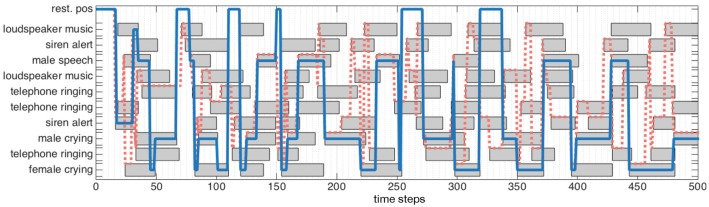
Audiovisual sources toward which a head movement has been triggered (blue line) by the dw module, (dotted red line) by the naive robot. (Gray boxes) audiovisual sources emitting sound. A source is focused on when the lines crosses the corresponding box.

Figure 4 exhibits one simulated environment, made of sources (represented as gray boxes) emitting sound along time (horizontal axis). Each source belongs to an audiovisual category represented on the left axis. Some sources might have the same audiovisual category: for instance, in this simulated scenario, the environment is made of three different telephones ringing. In addition to this “objects along time” description of the scene, Figure 4 shows two different lines: both “pass” through objects, indicating that the robot has decided to focus on them. The blue line corresponds to the decision taken by the dw module, while the red dashed one corresponds to ℜ_*n*_. Simulations show that the dw module considers the audiovisual classes (ringing, telephone) and (music, loudspeaker) as congruent in less than 100 time steps. This is because of their distribution with respect to the other categories: in the beginning of the simulation, objects belonging to these two categories are often present, making them less important. Consequently, the robot will not turn its head toward those sources: there is no (motor) attentional reaction anymore. On the other hand, the categories (alert, siren), (speech, male), (crying, female), and (crying, male) are considered as incongruent, thus requiring the robot to focus on them. Importantly, the actual meaning of those sources is not used here to decide of a reaction: one could have trade the congruent categories with the incongruent ones without any change in the global reaction. Only the frequency of apparition defined in Equation (5) is taken into account to decide the importance of a source.

**Figure 5:**
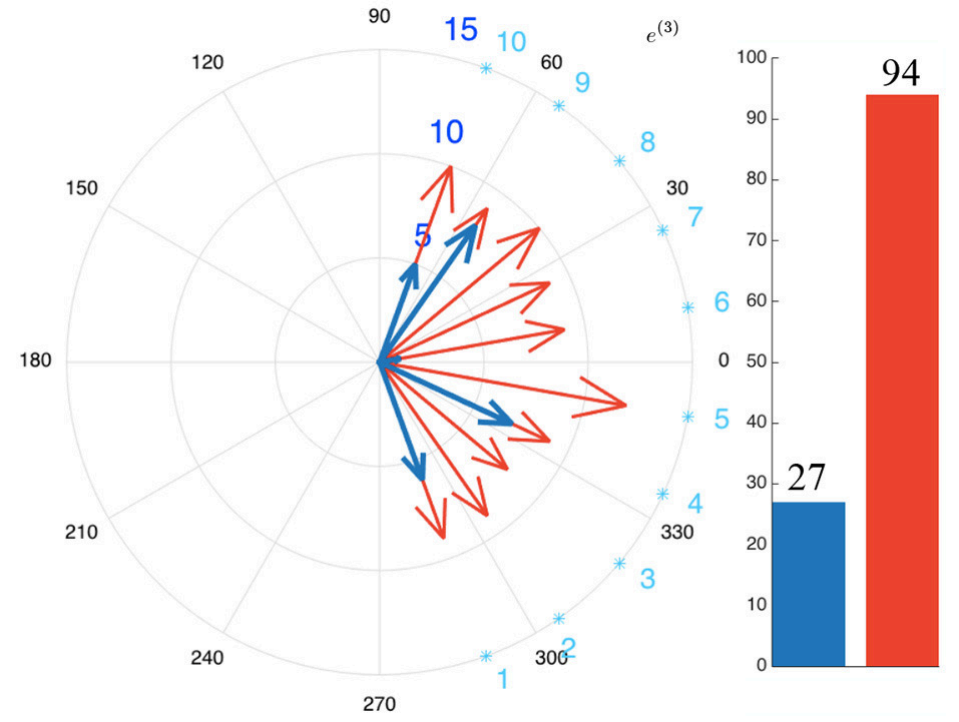
Head movements triggered by (blue) the dw module, (red) the virtual naive robot. Every arrow points toward the position of a source and their lengths depict the number of movements toward every pointed source. (Bars:) number of movements triggered by the the mfi module (blue) and the virtual naive robot (red). The light blue numbers correspond to the position of the audiovisual sources.

In comparison, the naive robot ℜ_*n*_ turns its head every time a source starts to emit sound: it is particularly noticeable between *t* = 200 and *t* = 250 where a lot of movements can be observed. The comparison between the two behaviors is highlighted in Figure 5, where is depicted the total number of head movements triggered to the audiovisual sources in the environment for the dw module (blue) and naive robot (red). It appears that a drastic modulation of the exploratory behavior is obtained: using the dw module conducts to a reduction of 71.3% of the number of head movements in comparison with the naive robot. Furthermore, the dw module only triggers movements toward five sources, instead of ten for the naive robot, thus showing how Congruence—even with its simple and intuitive definition—can provide an efficient filter for the attentional behavior of the robot. Importantly, such a modulation allows the robot to use head movements, and more generally its exploratory actions, for other unrelated tasks. As long as no incongruent source is detected, head movements are free to be used for anything else. But as soon as an incongruent source pops up in the environment, the dw module will drive the head toward this source: the robot then puts its attention on it. In the end, this simple illustration shows how important it is to be able to inhibit or trigger head movements.

#### 3.2.4. Conclusion and limitations

The dw module is a crucial part of the HTM system in charge with providing a semantic understanding of the unknown environments the robot is supposed to explore. One of the cornerstone of this module is to be able to work without prior knowledge about the potential distribution of the audiovisual sources occurring in these environments. Thus, the dw module has to create congruence rules on the sole basis of what the robot sees and hears, that is the audio and visual labels the classification experts output. The behavior rules created are, firstly, adaptive enough to always take into account new information, since the congruence status of all the objects are computed every time a new object is detected in the environment; and secondly, broad enough to limit any bias possible in the interpretation of the perceived information: an audiovisual class can be incongruent in an environment but congruent in another one, as will be illustrated in section 4. Moreover, by not creating any prior behavioral rules (such as *if-else* statements) and by letting the system continuously being sensitive to new information, the dw module provides the robot with a life-long learning of the environments composing the world it is living in. However, one important limitation appears here: the dw module needs to have access to a *complete* audiovisual information in order to compute the congruence of any object appearing in the scene. Indeed, in the situation where a source is placed behind the robot, it would have to first turn its head toward it in order to get the full audiovisual data, to then be able to take a decision on whether or not a head movement is necessary…which is what can be called a *deadlock* situation. This is why the previous illustration of the dw module has used a setup where all the visual data were always perceivable to the robot. Obviously, this is not a realistic context at all. This is where the second module of the HTM system comes into play.

### 3.3. The multimodal fusion and inference module

The *Multimodal Fusion & Inference* module (mfi module) is in charge of providing the dw module with a complete information about the audiovisual sources, even when they are placed behind the robot. Moreover, the mfi module is able to cope with classification errors, i.e., to provide a stable and reliable estimation of the audiovisual classes of an object. This module is based on an online self-supervised active learning paradigm that enables the overall system to create knowledge about the audiovisual classes that are present in the environments the robot is exploring. Basically, the idea is to exploit head movements to learn the relationship between the audio and visual classes of the sources, making the robot becoming afterwards able to infer a missing modality. To begin with, the learning paradigm of the mfi module is described in a first subsection. Then, the way motor orders are triggered to learn the association between audio and visual classes is presented. An illustration of the mfi module functioning together with new details concerning the simulations, are then provided. A short discussion ends this mfi module presentation.

#### 3.3.1. The multimodal self-organizing map

**Figure 6:**
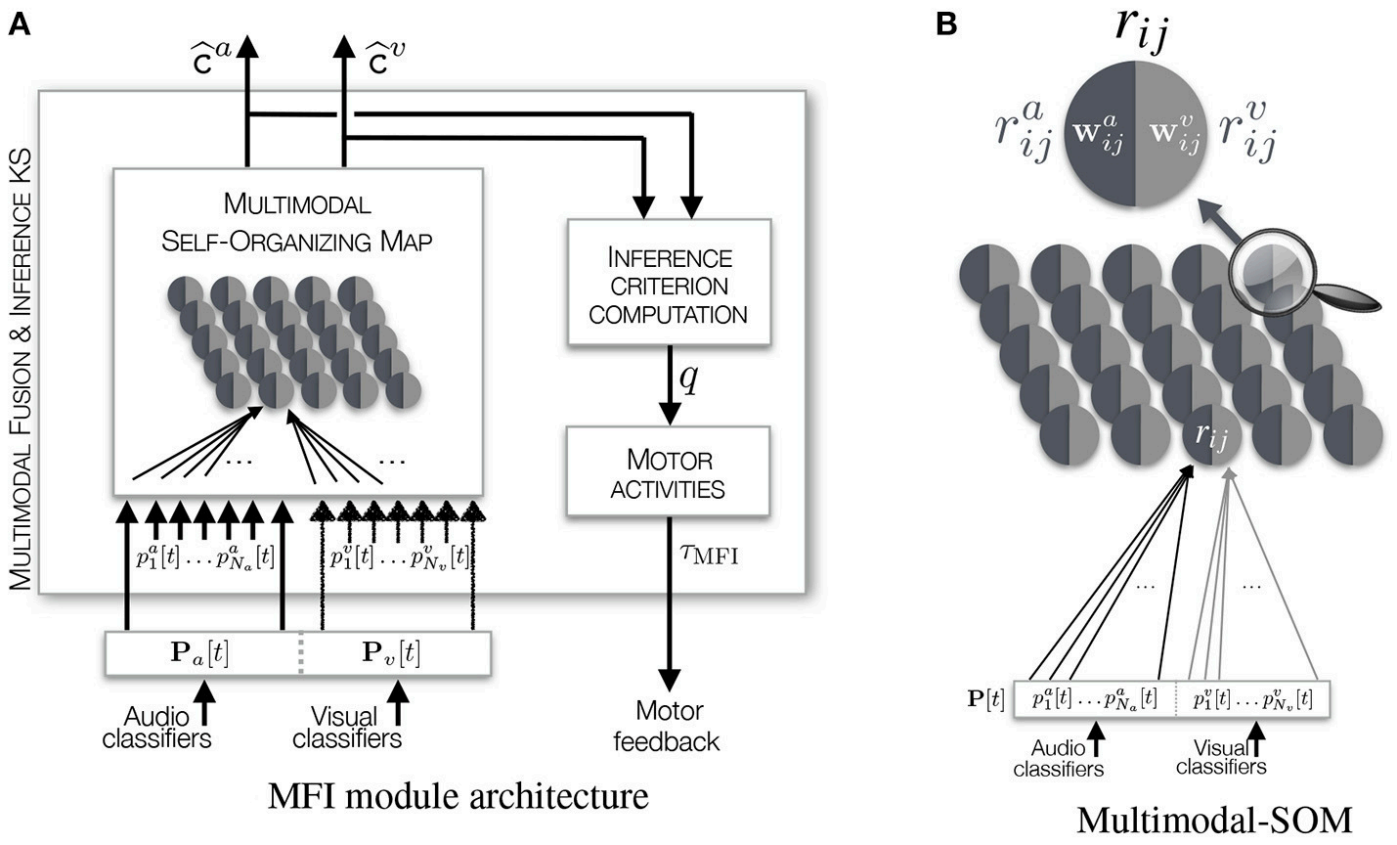
Illustration of **(A)** the Multimodal Fusion and Inference module, **(B)** the Multimodal Self- Organizing Map. The M-SOM embeds one SOM per modality used for the definition of an object (audio and vision in our case). The representation here depicts the two subnetworks as a map containing neurons split in two parts defined by their own weights vectors, one part being dedicated to the mapping of audio data, the other to visual data.

The mfi module is based on a Self-Organizing Map (SOM) Kohonen (1982) which is a learning algorithm relying upon a low dimensional map on which is performed a vector quantization of a high dimensional input matrix of data, while allowing its categorization. The input data are here made of classification experts outputs gathered in the vector **P**[*t*], see Figure 6A and Equation (1). However, the traditional SOM algorithm shows one important limitation: it is unable to cope with missing data. In the case where an event originates from behind the robot, visual classifier outputs will not be relevant: the visual modality is missing. Then, two options can be chosen: (i) remove the corresponding visual components of **P**[*t*], or (ii) set the corresponding components to the same arbitrary value. In the former case, this would imply a change in the data dimensionality. In the latter case, this would create arbitrary meaningful data which would be misinterpreted by the SOM. Then, these two options do not offer any solution to missing data inference. This is why it is proposed to transform a classical SOM into a *Multimodal*-SOM in order to keep what makes it powerful and usable with the constraints listed before. Interestingly, Papliński and Gustafsson (2005) have developed a bio-inspired system of interconnected SOMs allowing the learning of complex multimodal data for classification purpose. But while this system possesses interesting multimodal classification properties, it lacks the essential capability of inferring missing information. More recently, Bauer and Wermter (2013) and Schillaci et al. (2014) have proposed original models based on the SOM paradigm. But while they allow the multimodal learning of perceptual data in an unsupervised way, their major drawbacks reside either in their need of significant amount of data or in the time required to converge to a stable representation of the processed data.

##### 3.3.1.1. The subnetworks

Lets recall that a SOM is a map composed of *I*×*J* interconnected *r*_*ij*_ nodes, or neurons. The proposed modification of the original SOM consists in creating one SOM per modality, as shown in Figure 6B. Thus, the M-SOM is made of two (interdependent) maps, also composed by *I*×*J* interconnected $r_{ij}^{a/v}$ nodes, of size $\left\lceil \sqrt{N_{a}\times N_{v}}\right\rceil \times \left\lceil \sqrt{N_{a}\times N_{v}}\right\rceil$ (where *a*/*v* stands for *audio or visual* in a compact notation). This size has been selected to ensure that there will be at least one node available per possible audiovisual class combination, given that no prior information is available about the plausible audiovisual classes the robot will perceive during its life-long exploration. To each node is associated (i) a weights vector ${\text{w}}_{ij}^{a}=\left(w_{ij}^{a}\left(1\right),\ldots ,w_{ij}^{a}\left(N_{a}\right)\right)$ of size *N*_*a*_ for the audio subnetwork, and a weights vector ${\text{w}}_{ij}^{v}=\left(w_{ij}^{v}\left(1\right),\ldots ,w_{ij}^{v}\left(N_{v}\right)\right)$ of size *N*_*v*_ for the visual one, (ii) a (*i, j*) position in the map, and (iii) connections χ_(*ij*) → (*kl*)_ between the $r_{ij}^{a/v}$ nodes and their neighbors in the same map, where [*i, k*] ∈ [1, *I*] and [*j, l*] ∈ [1, *J*] (with an exception for the nodes located at the edges of the map where the connectivity is reduced). The weights vectors ${\text{w}}_{ij}^{a/v}$ associated to all the $r_{ij}^{a/v}$ nodes will become, through the iterative learning phase, the representatives of the different kinds of vectors constituting the input matrix, and thus, of the different audiovisual classes the input data capture.

##### 3.3.1.2. Weights update

Traditionally, at every iteration *n*_it_ of the original SOM algorithm (the total number of iterations classically going from thirty to thousands, given the complexity of the data to be processed), the input matrix is parsed randomly until every vector has been processed once (Kohonen, 2013). For every vector explored the algorithm looks then for the closest weights vector **w**_*ij*_ associated to the node *r*_*ij*_ to the current input vector, in terms of their Euclidean distance. The winning neuron, that is the one presenting the closest distance to the input vector, is called the *Best Matching Unit* (BMU). It will be the location in the map where the propagation of the resemblance between the input vector and the weights vector **w**_bmu_ will start. This propagation follows a Gaussian neighborhood function *h*_*ij*_[*n*_it_] (see Equation 14) of variance σ[*n*_it_] that defines the spread of the propagation. The neighborhood function is modulated by a factor α[*n*_it_], the learning rate, making the learning powerful in the first iterations but almost non-existent in the last ones. Spreading the resemblance to the BMU's neighbors has two effects: (i) lowering the distance between the BMU and the input vector so that this neuron becomes more and more the representative of the information coded by this vector, and (ii) partially shaping the map around the BMU so that the closest to the BMU in terms of distance, the closest also in terms of information coded by the input vector. This leads to an self-organized map where regions have emerged, regions that code for similar categories. Once every vector of the matrix has been explored, a new iteration of learning starts. At every iteration *n*_it_ is incremented making α[*n*_it_] and σ[*n*_it_] both decrease. Such decreasing leads to the following behavior of the learning process: at start, the propagation spreads largely in the SOM and the learning rate is at its highest; at the end of the learning, the propagation barely spreads around the BMU and the learning rate is at its lowest.

Within the M-SOM however, several changes of the traditional algorithm have been performed, changes that impact the way weights are updated. First, an audiovisual BMU $r_{\text{BMU}}^{av}$ is now computed as the combination of the two (audio and visual) subnetworks, according to

(12) $$\begin{array}{r} r_{\text{BMU}}^{av}=r_{IJ},\;\text{with}\left(I,J\right)=\arg\underset{i,j}{\min}\left(∥{\text{P}}^{a}-{\text{w}}_{ij}^{a}∥\times ∥{\text{P}}^{v}-{\text{w}}_{ij}^{v}∥\right), \end{array}$$

where ∥.∥ depicts the Euclidean distance between the vectors. This combined audiovisual BMU is associated to the combined weights vector ${\text{w}}_{\text{BMU}}^{av}=\left({\text{w}}_{\text{BMU}}^{a},{\text{w}}_{\text{BMU}}^{v}\right)$.

Secondly, the HTM does not have access to the whole matrix of data: the robot gets one vector at a time, every time a frame is analyzed by the set of KSs in the architecture. Thus, the iterative process has been revisited accordingly to this online paradigm. At every time step, the M-SOM will perform only 1 iteration of learning with the current vector (that is, there is no infinite memory of the past perceived data). However, the key principle of augmenting the resemblance between the BMU and the current vector, together with its spread, must be kept in order to reach an organized map. Taking also into account the fact that the audio classification experts from Two!Ears get more and more precise the longer they gather data from a same sound source, the evolution of α[*n*_*it*_] and σ[*n*_*it*_] has been reversed. The first steps of learning correspond to the minimum values of the learning rate and the variance of the neighborhood function, so that less importance is put to the very first classification experts data, and more to the next ones, following also the definition of an object (see section 2.2). Thirdly, still from the fact that the system does not have access to the whole data to be processed, it is necessary to adapt how the algorithm converges. Since the robot will always get to explore new environments during its life, there is no priorly known solutions to this learning problem. Consequently, instead of trying to reach a global convergence of the overall M-SOM, the MFI implements a *local consistency* (Chapelle et al., 2002; Zhou et al., 2004) at the audiovisual-class level (see also section 3.3.1.4). This local consistency enables the M-SOM to judge by itself whenever the learning of a particular class can be stopped or has to be continued. Thus, the value of the iteration *n*_it_, that will have an impact on the values of α and σ, will be computed *object by object*: every object has its own iteration value corresponding to a certain degree in the learning process of the audiovisual class it belongs to. The choice of implementing an iteration index object by object instead of class by class, which would seem more logical, comes also from the potentially erroneous behavior of the classification experts during the first perceived audio or visual frames associated to the objects (see section 2.2). Indeed, relying directly on these outputs could promote, on the mid- to long-term, the learning of false audiovisual classes that could hamper the learning of the correct ones. The learning iteration *n*_it_ is now defined by

(13) $$\begin{array}{r} \begin{array}{c} n_{\text{it}}\left[t\right]=\max\left(\left(N_{\text{it}}-n_{\text{it}}^{o_{j}}\left[t\right]\right)+1,1\right)\\ \text{with}n_{\text{it}}^{o_{j}}\left[t\right]=t_{\text{init}}\left(o_{j}\right)+\left(t-t_{\text{init}}\left(o_{j}\right)\right), \end{array} \end{array}$$

where *t*_init_(*o*_*j*_) is the temporal index corresponding to the initial time the object emitted sound in the current environment, and *N*_it_ = 10 corresponds to the maximal number of iterations. The value of *N*_it_ = 10 time steps has been defined experimentally with respect to two factors: (i) a too low value would put too much importance on the very first frames detected by the classifiers for a given object, and (ii) a too high value would significantly delay the local convergence of the learning for it would also delay the moment α and σ would be high enough to make the learning actually efficient.

Once $r_{\text{BMU}}^{av}$ is found, all the weights vectors associated with every node are then updated, as described above, and according to

(14) $$\begin{array}{r} \begin{array}{c} {\text{w}}_{ij}^{a/v}\left[t+1\right]={\text{w}}_{ij}^{a/v}\left[t\right]+\alpha \left[n_{\text{it}}\right]\;h_{ij}\left[t,n_{\text{it}}\right]\;∥{\text{P}}^{a/v}\left[t\right]-{\text{w}}_{ij}^{a/v}\left[t\right]∥,\\ \text{with}h_{ij}\left[t,n_{\text{it}}\right]=\exp\left(-\frac{∥r_{\text{BMU}}^{av}\left[t\right]-r_{ij}{∥}^{2}}{2\sigma {\left[n_{\text{it}}\right]}^{2}}\right), \end{array} \end{array}$$

where α ∈ [0.02, 0.9] represents the increasing learning rate (first and last values from Kohonen, 1990), and *h*_*i, j*_[*t, n*_*it*_] → ℝ is the Gaussian neighborhood function of variance σ[*n*_*it*_].

##### 3.3.1.3. Estimation of the audio and/or visual classes

Every time data **P**[*t*] are available from the KS, the M-SOM proposes a corresponding estimated audio and visual classes ${\hat{c}}^{a}$ and ${\hat{c}}^{v}$, respectively. In the case where all the data are available, then the corresponding classes can be estimated along

(15) $$\begin{array}{r} {\hat{c}}^{a}=c_{i}^{a},\;i=\arg\underset{l}{\max}w_{\text{BMU}}^{a}\left(l\right),\;\text{and }{\hat{c}}^{v}=c_{k}^{v},\;k=\arg\underset{l}{\max}w_{\text{BMU}}^{v}\left(l\right). \end{array}$$

Thus, the audiovisual class ${\hat{c}}^{\text{all}}$, estimated when all the modalities are available, is given by ${\hat{c}}^{\text{all}}=\left\{{\hat{c}}^{a},{\hat{c}}^{v}\right\}$. All the interest of the M-SOM is its ability to provide both audio and visual classes, even if a part the KS outputs are not available. Of course, no learning is then performed, but it is the step where the network is actually exploited for inference. In the case where, for instance, the visual data are missing (i.e., the event is out of the field of view of the robot), then:

1. audio only is exploited to determine the winning (audio) node $r_{\text{BMU}}^{a}$ in the audio map, whose associated weight vector ${\text{w}}_{\text{BMU}}^{a}$ can be used to estimate the audio class ${\hat{c}}^{a}=c_{i}^{a}$, with $i=\arg\underset{l}{\max}w_{\text{BMU}}^{a}\left(l\right)$ like in Equation (15);
2. the winning (visual) node is deduced from audio by $r_{\text{BMU}}^{v}=r_{\text{BMU}}^{a}$: this is exactly where the learned interlink between audio and visual data is exploited. The corresponding visual class ${\hat{c}}^{v}$ can then be deduced from the associated weight vector ${\text{w}}_{\text{BMU}}^{v}$ along ${\hat{c}}^{v}=c_{k}^{v}$, with $k=\arg\underset{l}{\max}w_{\text{BMU}}^{v}\left(l\right)$.

In the end, the audiovisual class ${\hat{c}}^{\text{miss}}$, estimated when one modality is missing, is then given by ${\hat{c}}^{\text{miss}}=\left\{{\hat{c}}^{a},{\hat{c}}^{v}\right\}$. Of course all the reasoning is identical when the other modality is missing: the available data drive the missing modality for inference.

##### 3.3.1.4. Convergence and the inference criterion

A key principle in learning algorithms is their ability to converge to one of the acceptable solutions of the problem to be solved. However in the proposed context, different environments made of possibly different audiovisual sources might be explored during the robot life. Then, it is clearly impossible to define one global good solution to the problem. Nevertheless, the proposed M-SOM possesses a characteristic of *local consistency* (see section 3.3.1.2). Within the classical SOM algorithm, convergence means that the whole map is organized such that the different nodes are grouped in meaningful entities that code part of the input data. In the proposed M-SOM, it is proposed that the algorithm always keeps a free part in the map, i.e., nodes not coding for any audio or visual classes. This would allow the network to include new audiovisual classes, discovered all along the interaction with new environments during the robot life. Looking for local consistencies, rather than reaching for global convergence, is implemented through the definition of a criterion for each audiovisual category already created, indicating how much this category has been learned so far and if its learning can be stopped. The multimodal learning performed by the mfi module is supported by head rotations to the sources to be learned. It allows to bring the visual sensors in front of them in order to learn the association between the corresponding audio and visual classes. But these head movements are no longer useful once the M-SOM has enough knowledge about the audiovisual classes, thus justifying the need to (i) inhibit these head movements, and (ii) being able to judge when this amount of knowledge is sufficient. Then, an *inference ratio* $q\left(C^{\left(l\right)}\left(c_{i}^{a},c_{k}^{v}\right)\right)$ for the audiovisual category $C^{\left(l\right)}\left(c_{i}^{a},c_{k}^{v}\right)$ is defined as

(16) $$\begin{array}{l} q(C^{\left(l\right)}({\text{c}}_{i}^{a},{\text{c}}_{k}^{v}))=\frac{\sum_{n=1}^{n=t}\delta_{i,k}^{\text{miss}}[n-1]\;\delta_{i,k}^{\text{all}}[n]}{\sum_{n=1}^{n=t}\delta_{i,k}^{\text{miss}}[n]},\\ \text{with}\delta_{i,k}^{\text{all/miss}}=\{\begin{array}{l} 1\text{ if }{\hat{c}}^{\text{all/miss}}(o_{j})=\{{\text{c}}_{i}^{a},{\text{c}}_{k}^{v}\},\\ 0\text{ else}. \end{array} \end{array}$$

This inference ratio is computed by comparing the number of times the audiovisual category $C^{\left(l\right)}\left(c_{i}^{a},c_{k}^{v}\right)$ has been obtained (or inferred) with one missing modality ($\delta_{i,k}^{\left(\text{miss}\right)}=1$) at time *n*−1 and confirmed at time *n* ($\delta_{i,k}^{\left(\text{all}\right)}=1$) by a head movement with all modalities available, with the total number of inference. Thus, $q\left(C^{\left(l\right)}\left(c_{i}^{a},c_{k}^{v}\right)\right)$ captures the ability of the mfi module to infer correctly a missing modality, category by category. The inference ratio always lies between 0 and 1, where 1 means that the category has always been perfectly inferred. On this basis, $q\left(C^{\left(l\right)}\left(c_{i}^{a},c_{k}^{v}\right)\right)$ is compared to a criterion $K_{q}\in R^{+}=\left[0,1\right]$: if a modality is missing, the mfi module will attempt to infer it, and as long as the inference ratio of the corresponding audiovisual category is lower than *K*_*q*_, a head movement will be triggered toward the corresponding source. Thus, the system grabs the missing information and feeds the M-SOM, which can then learn the audiovisual association. Of course, once the full audiovisual data is obtained, a comparison with the previous inference is made and the inference ratio is updated accordingly. If the inference ratio gets higher than the criterion *K*_*q*_, the learning is considered as being good enough to trust the inference made by the mfi module, and inhibit consequent head movements toward the sources belonging to the corresponding audiovisual category. Remark that the criterion *K*_*q*_ has an influence on the behavior of the mfi module (Cohen-Lhyver, 2017). A low threshold allows a quick confidence in the inference, thus freeing head movements for other tasks, whereas a high *K*_*q*_ value pushes the system to be very careful about its inferences.

#### 3.3.2. Motor orders

As for the dw module, the mfi module is able to trigger head movements toward sources of *interest*. This interest is now formalized by the lack of confidence in the knowledge of the audiovisual category a source might belong to. As previously explained, turning the head toward a source might enable the visual sensors to get the missing visual data, thus giving to the mfi module the opportunity to learn the interlink between the audio and visual modalities, but also to eventually confirm/refute an inference. Like for the dw module, the head movements modulation is inspired by the GPR model (see section 3.2.2), but through a different expression of the activities τ_mfi_(*o*_*j*_) for the object *o*_*j*_ with audiovisual category $C^{\left(l\right)}\left(c_{i}^{a},c_{k}^{v}\right)$, now given by

(17) $$\begin{array}{l} \tau_{\text{MFI}}(o_{j})=\frac{q(C^{\left(l\right)}({\text{c}}_{i}^{a},{\text{c}}_{k}^{v}))}{K_{q}}\times \delta^{\left(i,k\right)}(n),\\ \text{with }\delta^{\left(i,k\right)}(n)=\{\begin{array}{l} -1\text{ if }n<(t_{p}=10),\\ 1\text{ else}, \end{array} \end{array}$$

where *n* = *t* − *t*_foc_(*o*_*j*_), with *t*_foc_(*o*_*j*_) the first time the object has been focused on by the mfi module. Then, the angle $\hat{\theta }\left(o_{l}\right)$ estimated by the localization expert and corresponding to the canal with the lowest activity is selected as the winning motor order θ_mfi_, i.e.,

(18) $$\begin{array}{r} \theta_{\text{MFI}}=\hat{\theta }\left(o_{l}\right),\;\text{with }l=\arg\underset{j}{\min}\left(\tau_{\text{MFI}}\left(o_{j}\right)\right). \end{array}$$

**Figure 7:**
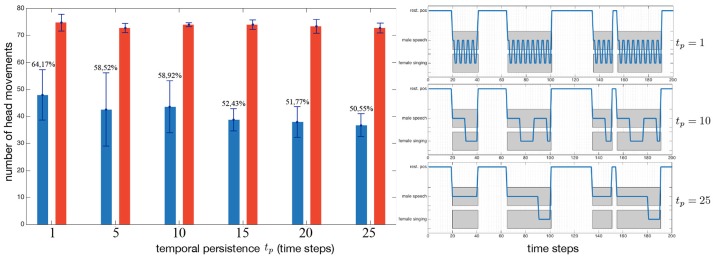
Impact of the temporal persistence, introduced in Equation (17), (left) on the number of triggered head movements in a complex environment, and (right) the behavior of the robot in a simplified case for illustration purposes. (Blue bars:) robot driven by the mfi module, (red bars) naive robot. Percentages depict the ratio between the naive robot and the mfi module.

The term δ^(*i, k*)^(*n*) in (17) introduces a form of temporal persistence through a positive feedback loop, as observed in the thalamus by Redgrave et al. (1999), Gurney et al. (2001a), and Meyer et al. (2005). The value of *t*_*p*_ = 10 has been set experimentally after several comparisons and evaluations. The impact of this persistence in a complex environment (eight sources with five simultaneously emitting) is illustrated in the left panel of Figure 7, where the blue bars depict the number of head movements triggered by the mfi module, while the red bars, by the naive robot ℜ_*n*_ (these numbers are obviously not affected by the temporal persistence applied to the mfi module). The main point is that the temporal persistence *t*_*p*_ constitutes only a small part of the head movements control: 13.6% less head movements between *t*_*p*_ = 1 and *t*_*p*_ = 25. The real benefits of temporal persistence is shown in Figure 7 (right): with *t*_*p*_ = 1, the robot exhibits oscillations between two sources, potentially damaging the internal representation of the world (confusions in binaural cues computations, speed of the movement…). With *t*_*p*_ = 25, a pervert effect of a too long persistence is also shown: the system often ghosts completely the (singing, female) source, preventing itself from learning it.

#### 3.3.3. Evaluation 2: classification rates of the mfi module

The mfi module aims providing a corrected audiovisual information from the classification experts. In order to assess the contribution brought by this module, a good audiovisual classification rate **Γ**(*o*_*j*_)[*t*] is defined by comparing the audio and visual classes associated to all the objects detected by the system with the ground truth, according to

(19) $$\begin{array}{ll} \Gamma (o_{j})[t] & =a\times \sum_{k=\;t_{i}}^{t}\gamma (o_{j})[k]\\ \text{with }\gamma (o_{j})[k]=\{\begin{array}{l} 1\text{ if}\hat{\;c}(o_{j})[k]=c(\psi_{j})[k],\\ 0\text{ else}, \end{array} \end{array}$$

with *c*(Ψ_*j*_)[*k*] being the ground truth audiovisual class of the event Ψ_*j*_ captured as the object *o*_*j*_ at time *k* in the internal representation, and **a** = 1/[1, …, (*t* − *t*_*i*_) + 1] corresponding to the elapsed time between the first time step *t*_*i*_ when the mfi module provided a classification of the object *o*_*j*_, and the current time *t*. The overall good classification rate is given by applying a sliding window on all **Γ**(*o*_*j*_) computed from the beginning of the exploration, along

(20) $$\begin{array}{r} {\bar{\Gamma }}_{\text{MFI}}\left[t\right]=\frac{1}{N_{obj}^{c}\left[t\right]}\overset{N_{obj}^{c}\left[t\right]}{\underset{j=1}{\sum }}\Gamma \left(o_{j}\right)\left[t\right] \end{array}$$

with $N_{obj}^{c}\left[t\right]$ the number of processed objects by the mfi module at time *t* (this number could be inferior or equal to the total number of objects present and emitting, noted *N*_*obj*_). In parallel, the same process is made for the naive robot ℜ_*n*_ [knowing that this one performs the fusion of the classification experts themselves through a maximum a posteriori approach, along Equation (11)], according to

(21) $$\begin{array}{r} {\bar{\Gamma }}_{{ℜ}_{n}}\left[t\right]=\frac{1}{N_{obj}\left[t\right]}\overset{N_{obj}\left[t\right]}{\underset{j=1}{\sum }}\Gamma \left(o_{j}\right)\left[t\right]. \end{array}$$

In addition, a measure of the classification performance of an omniscient (thus unrealistic) robot is also computed, noted ${\bar{\Gamma }}_{{ℜ}_{n}}^{\prime }\left[t\right]$. This robot has full access to every auditory and visual information, even when the objects are out of the sight of the robot. The simulation setup is presented in Table 1. Twenty different multisource environments are simulated, each of them in possibly different conditions (number of sources, number of simultaneously emitting sources, error rates, etc.). The resulting good audiovisual classification rates are regrouped in Table 2, mainly organized by increasing error rates $\varepsilon_{P}=\left\{0.1,0.3,0.5,0.7,0.9\right\}$.

**Table 1:** Simulation setup for Evaluation 2.

**Evaluation 2**						
***e*^(*l*)^**	**$n_{S}$**	**$n_{sim}^{max}$**	***T***	**$\left|C^{\left(l\right)}\right|$**	***K*_*q*_**	**ε_P_**
1 to 5	3	3	1000	2	0.8	0.1, 0.3, 0.5, 0.7, 0.9
6 to 10	5	5	1000	3	0.8	0.1, 0.3, 0.5, 0.7, 0.9
11 to 15	7	7	1000	4	0.8	0.1, 0.3, 0.5, 0.7, 0.9
16 to 20	10	10	1000	6	0.8	0.1, 0.3, 0.5, 0.7, 0.9

*Each setup is repeated 5 times for a total of 100 simulations*.

**Table 2:** Good classification rates for different error rates and different numbers of sources.

**Evaluation 2: Results**					
ε_P_	$n_{S}$ **—** $n_{sim}^{max}$	${\bar{\Gamma }}_{\text{MFI}}\left[t=T\right]$	${\bar{\Gamma }}_{{ℜ}_{n}}^{\prime }\left[t=T\right]$	${\bar{\Gamma }}_{{ℜ}_{n}}\left[t=T\right]$	**ratio:** $\frac{{\bar{\Gamma }}_{\text{MFI}}\left[t=T\right]}{{\bar{\Gamma }}_{{ℜ}_{n}}^{\prime }\left[t=T\right]}$
0.1	3 — 3	0.982 (0.027)	0.894 (0.021)	0.503 (0.073)	1.098
	5 — 5	0.988 (0.025)	0.899 (0.012)	0.339 (0.039)	1.099
	7 — 7	0.960 (0.023)	0.893 (0.016)	0.264 (0.021)	1.075
	10 — 10	0.866 (0.047)	0.887 (0.018)	0.182 (0.014)	0.976
	Mean	0.949	0.893	0.322	1.063
0.3	3 — 3	0.992 (0.020)	0.703 (0.042)	0.414 (0.055)	1.411
	5 — 5	0.987 (0.022)	0.692 (0.017)	0.265 (0.014)	1.426
	7 — 7	0.942 (0.028)	0.691 (0.014)	0.198 (0.017)	1.363
	10 — 10	0.883 (0.041)	0.689 (0.011)	0.145 (0.014)	1.281
	Mean	0.951	0.693	0.255	1.372
0.5	3 — 3	0.973 (0.026)	0.493 (0.020)	0.280 (0.031)	1.973
	5 — 5	0.965 (0.043)	0.496 (0.021)	0.189 (0.034)	1.945
	7 — 7	0.899 (0.048)	0.492 (0.018)	0.145 (0.019)	1.827
	10 — 10	0.836 (0.042)	0.492 (0.018)	0.103 (0.010)	1.699
	Mean	0.918	0.493	0.179	1.862
0.7	3 — 3	0.774 (0.087)	0.282 (0.030)	0.165 (0.028)	2.744
	5 — 5	0.737 (0.105)	0.294 (0.014)	0.120 (0.023)	2.506
	7 — 7	0.683 (0.133)	0.296 (0.016)	0.081 (0.012)	2.307
	10 — 10	0.550 (0.117)	0.293 (0.016)	0.064 (0.011)	1.877
	Mean	0.686	0.291	0.107	2.357
0.9	3 — 3	0.213 (0.060)	0.092 (0.019)	0.054 (0.019)	2.315
	5 — 5	0.152 (0.064)	0.102 (0.012)	0.039 (0.007)	1.490
	7 — 7	0.174 (0.075)	0.100 (0.009)	0.031 (0.005)	1.740
	10 — 10	0.140 (0.066)	0.100 (0.009)	0.019 (0.006)	1.400
	Mean	0.169	0.098	0.035	1.724

*Every results is an average of 5 repetitions of every conditions with standard deviation in parentheses, for a total of 100 simulations. ℜ_n_ corresponds to the naive robot, and ${ℜ}_{n}^{\prime }$ to the unrealistic omniscient robot. Values are rounded up to the third decimal*.

At first, let us consider the naive omniscient robot ${ℜ}_{n}^{\prime }$. As expected, it presents a mean good audiovisual classification rate ${\bar{\Gamma }}_{{ℜ}_{n}^{\prime }}$ almost equal to $1-\varepsilon_{P}$ for all tested conditions. In contrast, the realistic naive robot ℜ_*n*_ (having only access to the data it is able to perceive) systematically exhibits lower rates ${\bar{\Gamma }}_{{ℜ}_{n}}$. Clearly, the main flaw of this robot is its incapacity to perform any inference, which turns to be a critical capability in multisource environments. In comparison, the proposed mfi module outperforms both naive robots, for almost any error rates and number of sources (except for only one case: $\varepsilon_{P}=0.1$ and $n_{S}=10$). The last column in Table 2 exhibits the ratio between the best naive robot ℜ_*n*_ (given by ${\bar{\Gamma }}_{{ℜ}_{n}}^{\prime }\left[t=T\right]$) and the mfi module: the greater $\varepsilon_{P}$, the higher the ratio, except with $\varepsilon_{P}=0.9$. In this case, the error rate is anyway so high that the interest in exploiting such corrupted data is almost null. However, even in very challenging conditions involving a very high $\varepsilon_{P}=0.7$ in a multisource context, the mfi module provides on average a 2.4 times better good audiovisual classification rate than with the classifier outputs.

#### 3.3.4. Discussion

The proposed mfi module, mainly based on the M-SOM, provides an online self-supervised active learning paradigm to be able to process erroneous and/or missing data in the particular context of the exploration of unknown environments. The overall goal of the mfi module is thus to feed the dw module with correct audiovisual classes the perceived objects belong to, with respect to a very short learning time constraint (down to a few seconds only). The *active* capabilities of the mfi module is of very much importance here, for it enables the intensive use of head movements to gather, whenever it is necessary, and in real-time, additional data to refine the knowledge the module has of the world under exploration. A fundamental question arises with the problem of audio and visual classes association when considering one-to-one audiovisual pairs, i.e., that each audio label is associated with only one visual label, and vice and versa. In the evaluations presented in this section, such pairing limitation was not used: an audio label could have several visual correspondences, such as speaking, male, speaking, female, or speaking, child. However, given these audiovisual labels examples, it is not possible for the mfi module to create an information that does not exist: from the audio label speaking, it is impossible to determine whether the corresponding visual label is male, female, or child. The mfi module still outputs an hypothesis corresponding, given how the M-SOM learning algorithm works, to the most observed so far audiovisual pair. Such limitation of the mfi module only comes from the limits of the classification experts themselves: if the classifiers cannot distinguish a female voice from a male one, nor would the mfi module. Such a case will be shown and also discussed in section 4.4, when evaluating the whole system in real environments.

### 3.4. Conclusion

The Head Turning Modulation system is composed with two modules: the Dynamic Weighting module (DW) and the Multimodal Fusion and Inference module (MFI), each of them having been described in this section. The dw module is an attentional component, working on the sole basis of observed data in unknown environments, from which it enables the robot to turn its head to audiovisual sources considered as “of importance.” Coupled to it is the mfi module that learns the relationship between the modalities that are used to define an object (audition and vision in this case). Based on a Multimodal Self-Organizing Map (M-SOM), the mfi module is able to create the knowledge required by the dw module to work properly. This knowledge consists in the fusion of multimodal data into a corrected database of audiovisual categories, knowledge that is created through online active self-supervised exploration of the audiovisual sources appearing in the unknown environments. Both modules can trigger head movements independently, and their combination necessitates an adaptation of the motor orders expressions of the modules.

The next section will present the results obtained in real environments with the real robot embedding the whole Two!Ears software (including the integration of the HTM system), and processing real audio and visual data.

## 4. Combination of the two modules

The previous section was dedicated to the individual presentation of each module constituting the HTM system, while providing limited evaluations in simulated conditions. This section is now concerned with the combination of the dw module and the mfi module together, with their evaluation in realistic conditions, i.e., on a real robot and with real audio and visual data. At first, one have to deal with the fact that theses two modules are both able to generate competitive head movements. The way they are prioritized is described in a first subsection. Next, the experimental setup is carefully described in a second subsection. Then, experimental results are provided in a third subsection, aiming at demonstrating the benefits of the overall system in the audiovisual scene understanding.

### 4.1. Combined motor orders: evaluation 3

It has been shown in section 3 that the dw module and the mfi module both exploit head movements to better their respective operations. Trying to make them able to work together then requires a prioritization of them. On the one hand, the dw module provides the robot with potential sources to be focused on, on the basis of their computed congruence; on the other hand, the mfi module aims at estimating audiovisual classes of objects inside the environment, even with potential classification errors and lack of data. It seems then obvious to set the priority to the mfi module: having a reliable audiovisual classes estimation system is required for the attentional module to take relevant decisions. This prioritization introduces a new activity $\tau_{\text{DW}}^{\prime }$ for the dw module which is now defined, for an object *o*_*j*_, by

(22) $$\begin{array}{l} {\tau^{\prime }}_{\text{DW}}(o_{j})=\tau_{\text{MFI}}(o_{j})-\tau_{\text{DW}}(o_{j})\times \delta (\tau_{\text{MFI}}(o_{j})),\\ \text{with }\delta (x)=\{\begin{array}{l} 1,\text{ if }x\geq 1,\\ 0,\text{ otherwise}. \end{array} \end{array}$$

**Figure 8:**
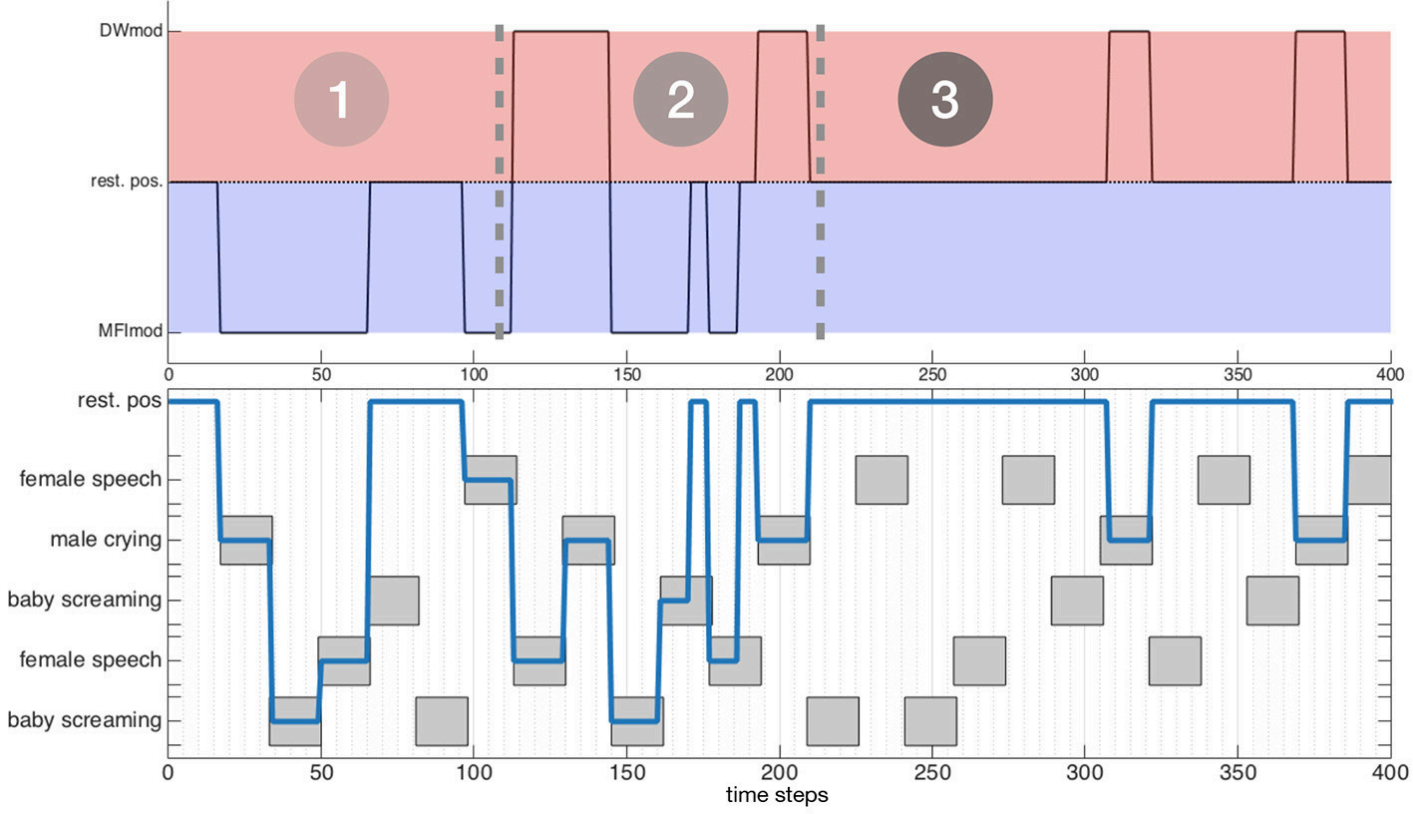
Behavior of the combined modules in three phases. The testing environment is composed of five audiovisual sources and is willingly simple for illustration purposes. **(Top)** Module ordering the head movement. **(Bottom)** Temporal course of the exploration of the environment; gray boxes depict the temporal course of emitting sources.

On this basis, the motor order θ_htm_ selected to drive the head is computed along

(23) $$\begin{array}{l} \theta_{\text{HTM}}=\hat{\theta }(o_{l}),\text{ with }l=\arg_{j_{1},j_{2}}\min({\tau^{\prime }}_{DW}(o_{j_{1}}),\tau_{\text{MFI}}(o_{j_{2}}))\\ \text{where }\{\begin{array}{c} j_{1}=\arg\;\min_{l}({\tau^{\prime }}_{\text{DW}}(o_{l})),\\ j_{2}=\arg\;\min_{k}(\tau_{\text{MFI}}(o_{k})), \end{array} \end{array}$$

i.e., the object with the lowest dw module or mfi module activity is selected. Such a modification of the motor activity expression enables the mfi module to take over the lead on the dw module. The evaluation of such a modification in the motor commands decision system can be performed again in simulation, along the same procedure as in the previous simulations, see Figure 8. Let us consider an environment made of five objects, belonging to three different audiovisual categories. Each of these objects emit sounds along time, according to the time plot shown in Figure 8 (bottom). Figure 8 (top) exhibits the three-phase behavior of the motor decision algorithm. At the very beginning, only the mfi module is responsible for the head movements: the system is learning the association between audiovisual classes. Little by little, the inference provided by the mfi module does not need motor confirmation for some of the classes: the dw module can now compute congruence of the corresponding objects and potentially trigger head movements. In the end, all the audiovisual classes are correctly learned by the mfi module, letting the sole dw module in charge with head rotations. Of course, the head movements triggered by the dw module are also used to feed the M-SOM.

### 4.2. Experimental setup and data generation

**Figure 9:**
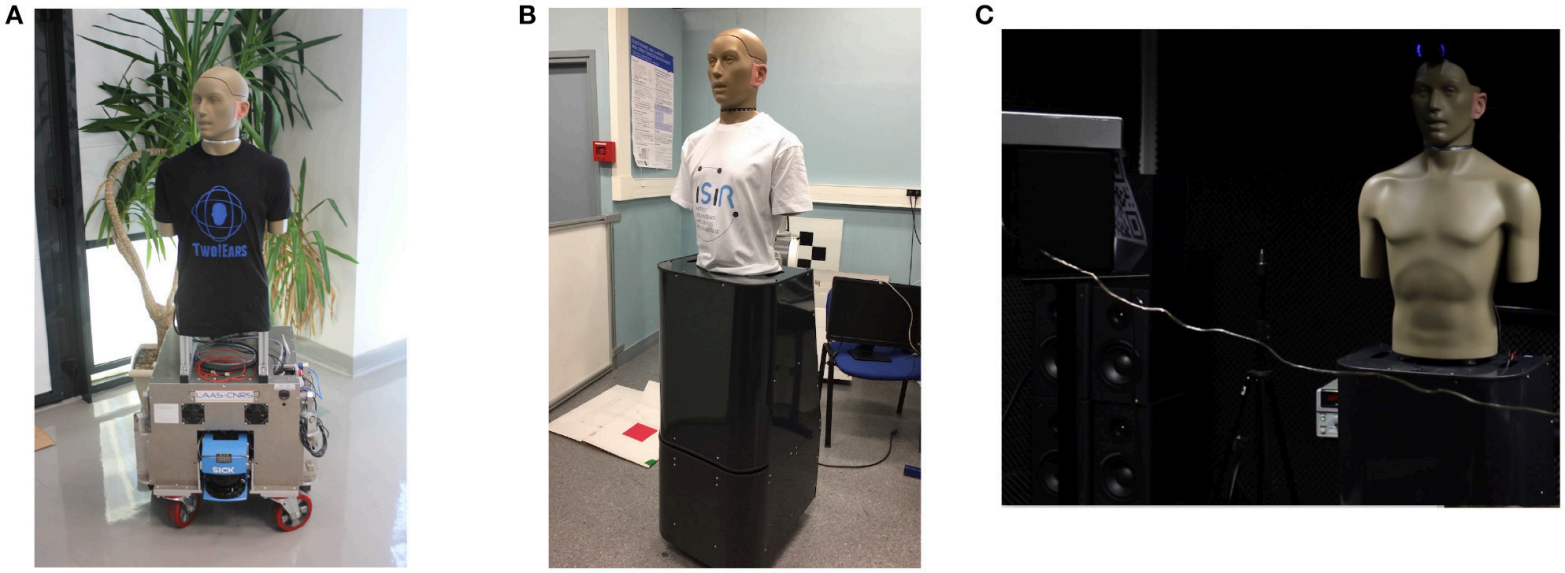
The two robotic platforms used in the project, both supporting a motorized KEMAR HATS. ODI has been used in this paper for the HTM evaluation. **(A)** The ODI platform. **(B)** The ODI platform. **(C)** ODI, facing a loudspeaker with a QR code attached on it.

The overall system has been evaluated in a realistic environment by using a real robot integrating the whole Two!Ears software and evolving in a real room. In practice, two different robots have been actually used: one mobile platform from LAAS-CNRS (Toulouse, France) named Jido, the other one from ISIR (Paris, France) named Odi, see pictures in Figure 9. Both platforms support a KEMAR HATS (Head And Torso Simulator), whose necks have been motorized to control their head movements in azimuth (Bustamante et al., 2016). A HATS is a manikin endowed with two microphones placed inside two pinnae which mimics the acoustic effect of the head (and torso) on the left and right ear signals, thus producing a realistic binaural information, close to what a human could actually hears. The servo control of the head is ensured by a set including a motor, its gear head, an encoder, and an Harmonica electronic controller from ELMO, mounted inside the HATS. A ROS node dedicated to the head control is in charge of controlling this motorization, allowing real-time servoing of the head movements by using possibly different feedback control options like position or velocity setpoints. In this paper, the positions deduced from Equation (23) are directly sent to the ROS node to control the head in position. These two robots are very much alike, except for vision: the one used at ISIR for the experiments used in this paper is only endowed with monocular vision. However, as already argued, the HTM system is not dependent on the way each modality works, but only on the identification experts, be they dedicated to monocular or binocular vision for instance.

Everything related to the platform and data acquisition is handled by the ROS middleware, running directly on the robot: navigation, obstacle avoidance, image and audio captures, etc. Note that a dedicated ROS binaural processing node has been developed during the project, so that most of the audio cues required for sound localization, recognition and separation are directly computed in real-time on the robot. State-of-the-art ROS nodes dedicated to vision (acquisition and processing) have also been used. All the data computed on the robot are then transmitted to another computer running the Two!Ears framework thanks to a MATLAB-to-ROS bridge. This bridge has been entirely designed to deal with the proposed bottom-up and top-down approach of the project, so that all the ROS nodes can be easily parameterized on the fly and in real time. Then, all the steps required for the “cognitive” analysis (i.e., object localization, recognition, fusion, etc.) runs under MATLAB.

Experiments used in this paper have been conducted in a pseudo-anechoic room populated with loudspeakers over which QR codes have been attached to, see Figure 9C. These are used by a ROS node to extract the visual labels of each object directly and with a recognition rate similar to the one obtained through the binocular vision of Jido with the Line-Mod algorithm Hinterstoisser et al. (2012). All the sounds emitted from the loudspeakers belong to a database constituted of sounds used to train the audio experts in recognition. In other terms, all the sounds can be recognized by at least one expert in the architecture. Then, the HTM has been evaluated in experimental conditions by two scenarios: the first emphasizes the global behavior of the system, while the second focuses on the fusion and classification abilities of the mfi module. Whatever the scenarios, they all works the following way: sounds are emitted from one or multiple loudspeakers, possibly at the same time. Depending on how the head of the KEMAR is turned, some QR codes can be manually changed from one loudspeaker to another to simulate an object movement in the environment. The HTM system then gathers classification and localization results coming from the audio and visual experts, and triggers some head movements accordingly. A scenario is entirely described by the number of different objects in the scene and by the time description of their localization, appearance and disappearance, exactly like in the previous simulations. Of course, ground truth audio and visual classes of each object are known, thus allowing a careful evaluation of the overall system performance. Note that the audio experts used in the following experiments have been set up by using data from a database recorded in a different acoustic environment. Since they all rely on a prior learning step exploiting these data, there will be a mismatch between their learning and testing phase. The main consequence is mainly a lower frame recognition rate, evaluated to about 37% for the four classifiers used here, and that have been chosen amongst the most performing ones (Two!Ears, 2016a). The same applies to the localization algorithm, with less consequences: experiments still show a good ability to localize sounds with a precision of about 7.7° (including front-back confusion). Finally, the visual recognition of QR codes works almost perfectly, while being quite sensitive to changes in illuminations. Of course, both phenomena are dealt with the HTM system, which has been entirely designed to cope with recognition errors and lack of data, as show in the next subsections.

### 4.3. Evaluation 4: global behavior

**Figure 10:**
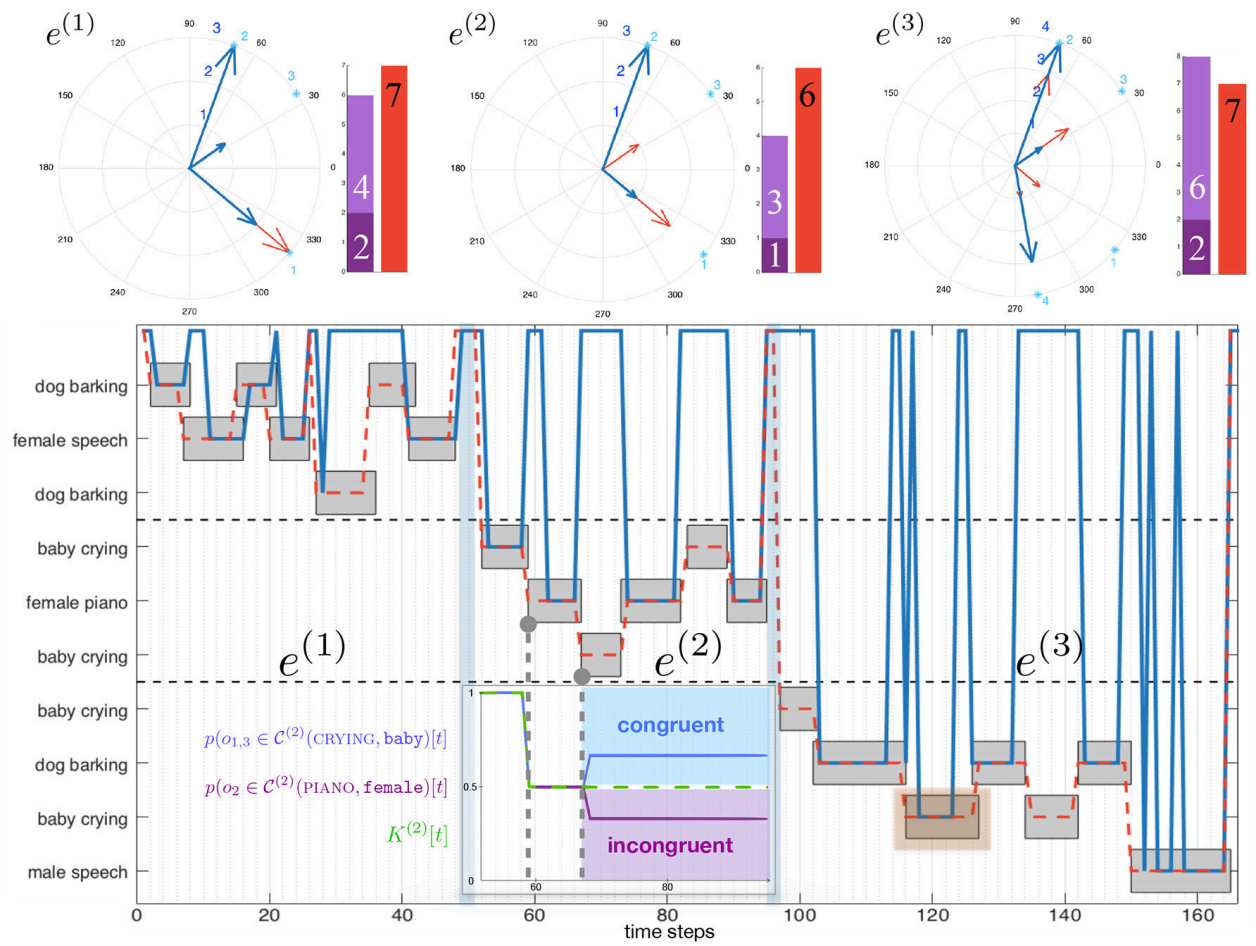
**(Top)** Number of head movements triggered during the exploration of each environment by (blue) the HTMKS, (red) the virtual naive robot. Each arrow points at a source and their length represent the number of movements. The light blue numbers correspond to the position of the sources. (Purple bars:) total number of movements triggered by (dark) the mfi module, (light) the dw module (black numbers are their sum). (Red bars:) number of movements triggered by the virtual naive robot. **(Bottom)** Movements triggered by (blue line) the HTMKS, and (dotted red line) the naive robot. (Gray boxes:) temporal course of the scenarios. The semi-transparent red box at t = 116 highlights the significant wrongful discrepancy that occurred between the actual audiovisual class of the object and the perception of the HTM (error that is corrected soon after, see text for more details). Additionally, the subfigure present in the delineated box at the bottom of *e*^(2)^ represents the evolution of *K*^(2)^ together with the posterior probabilities of the two audiovisual classes observed in *e*^(2)^ (in light blue for *o*_1_ and *o*_3_, in purple for *o*_2_). The comparison of the all the *p*(*o*_*j*_) and *K*^(*l*)^ justifies the potential triggering of head movements by the dw module, as observed at *t* = 74 and *t* = 90.

This first evaluation aims at demonstrating how the two modules constituting the HTM system cooperate together in order the exploratory robot an additional understanding of the world. The evaluation consists in presenting to the system three successive environments made of three to four objects, as summarized in Table 3. The audiovisual sources of the environments are placed around the robot and emit sound intermittently, according to the time scenario shown in Figure 10 (bottom). Exactly like in simulations, the real robot is compared to its naive counterpart ℜ_*n*_, turning its head toward every audiovisual events regardless of their meaning. To begin with, the HTM builds a first representation *e*^(1)^. As shown in Figure 10, the robot starts by turning its head toward the first two audiovisual sources (barking, dog and speech, female), driven by the mfi module since these audiovisual classes are brand new to it. As already outlined in the previous subsection, the HTM tries to learn the audiovisual association between these two classes. This learning is done very quickly: one can observe at time index *t* = 28 (corresponding to the “real” time 14 s) that the robot turns its head to its resting state (blue line going at the top of the figure), meaning that neither the dw module nor the mfi module requires a head movement toward the sources barking, dog: these sources are not of interest anymore, and hearing the sound barking is sufficient to infer the visual class dog. Nevertheless, one can remark a glitch in the head movement decision at *t* = 30, as the last attempt of the mfi module to learn the barking, dog audiovisual association. At *t* = 41 (20.5 s), the robot turns its head again toward the source (speech, female): with two barking, dog for one speech, female in *e*^(1)^, the probability for this last audiovisual category $p\left(C^{\left(1\right)}\left(\text{SPEECH},\text{female}\right)\right)=1/3$ falls below *K*^(1)^ = 1/2, thus making any object of this audiovisual category incongruent. Then, the robot explores a second environment. It is similar in terms of frequencies of apparition of each audiovisual categories, even if their meaning (at least, to us) is different: the two barking, dog are trade for two crying, baby, while the category speech, female is replaced by piano, female. Logically, the obtained behavior is similar: a quick learning of the audiovisual association allows then the head to be controlled by the dw module on the basis on congruency computations. Interestingly, the understanding of this second environment by the dw module could appear as counterintuitive in comparison with how humans might have reacted by favoring the two objects crying, baby. This more social reaction could nevertheless be handled by some additional KS from the Two!Ears architecture which could modulate the overall reaction of the robot w.r.t. the current task (Ferreira and Dias, 2014). Finally, a third environment is explored. It will allow to demonstrate the benefits of reusing information between the representation of environments, see section3.2.1. Indeed, the scene begins with a crying, baby which does not trigger any head movement: while being in a new environment, the HTM system considers at this point that this third environment is very likely to be the same as *e*^(2)^ where this audiovisual class was considered as congruent. Consequently, the congruence computations of each audiovisual categories in the previous environment can still be used, and no head movements toward this now object is performed. However, as soon as a new object eliminates the possibility to be in an environment similar to *e*^(2)^ pops up, a new representation *e*^(3)^ is created. Thus, when the source barking, dog appears in the scene, a head movement is immediately triggered toward it, since it is incongruent in *e*^(3)^. Once again, a small glitch in the motor decision appears in *t* = 116, caused by the experts outputs and the signal non-stationarity (a barking sound includes indeed some silence). The movement triggered at *t* = 118 is an error from the system since the object crying, baby should have been considered as congruent. The audio data perceived at this time is maleSpeech, data from a never encountered audio class, thus enjoining the mfi module to trigger a head movement. From *t* = 119 and *t* = 122, the experts' data changed and became of class crying, dog, an audiovisual pair the mfi module never encountered before, consequently still promoting the focus on the object. However, at time *t* = 123, the correction of the mfi module has been applied and the “correct” audiovisual class crying, baby is now output by the module. The dw module, in response, analyses it and consider it as congruent in this environment, thus inhibiting the head movement. Finally, the new source speech, male appears in the environment at *t* = 150 and the robot is focused on it. Two (apparently) erroneous movements to the resting position can be observed, at *t* = 153 and *t* = 157, due to the discontinuity of the sound signal: the audio experts did not detect any sound for these two frames (to give an idea: argmax(**P**^*a*^[*t* = 157]) = 0.176, whereas for the frame right before, at *t* = 156, five components out of thirteen are lying between *p*^*a*^ = 0.403 and *p*^*a*^ = 1.00). Going back to the resting position when an object stops emitting sound is part of the attempt of the overall HTM system to also inhibit the head movements in order to free the head for other potential purposes.

**Table 3:** Experimental setup for Evaluations 4 & 5.

***e*^(*i*)^**	**$n_{S}$**	**$n_{sim}^{max}$**	***c*(Ψ_*j*_)**	**θ(Ψ_*j*_)**	***K_q_***
**EVALUATION 4**					
1	3	1	*dog barking* n°1	320°	0.6
			*dog barking* n°2	35°	
			*female speech*	70°	
2	3	1	*baby crying* n°1	70°	0.6
			*baby crying* n°2	35°	
			*female piano*	320°	
3	4	1	*baby crying* n°1	70°	0.6
			*baby crying* n°2	35°	
			*dog barking*	320°	
			*male speech*	280°	
**EVALUATION 5**					
1	5	1	*female speech*	320°	0.6
			*female piano*	30°	
			*male speech*	60°	
			*dog barking*	90°	
			*baby screaming*	280°	

### 4.4. Evaluation 5: fusion and classification

**Figure 11:**
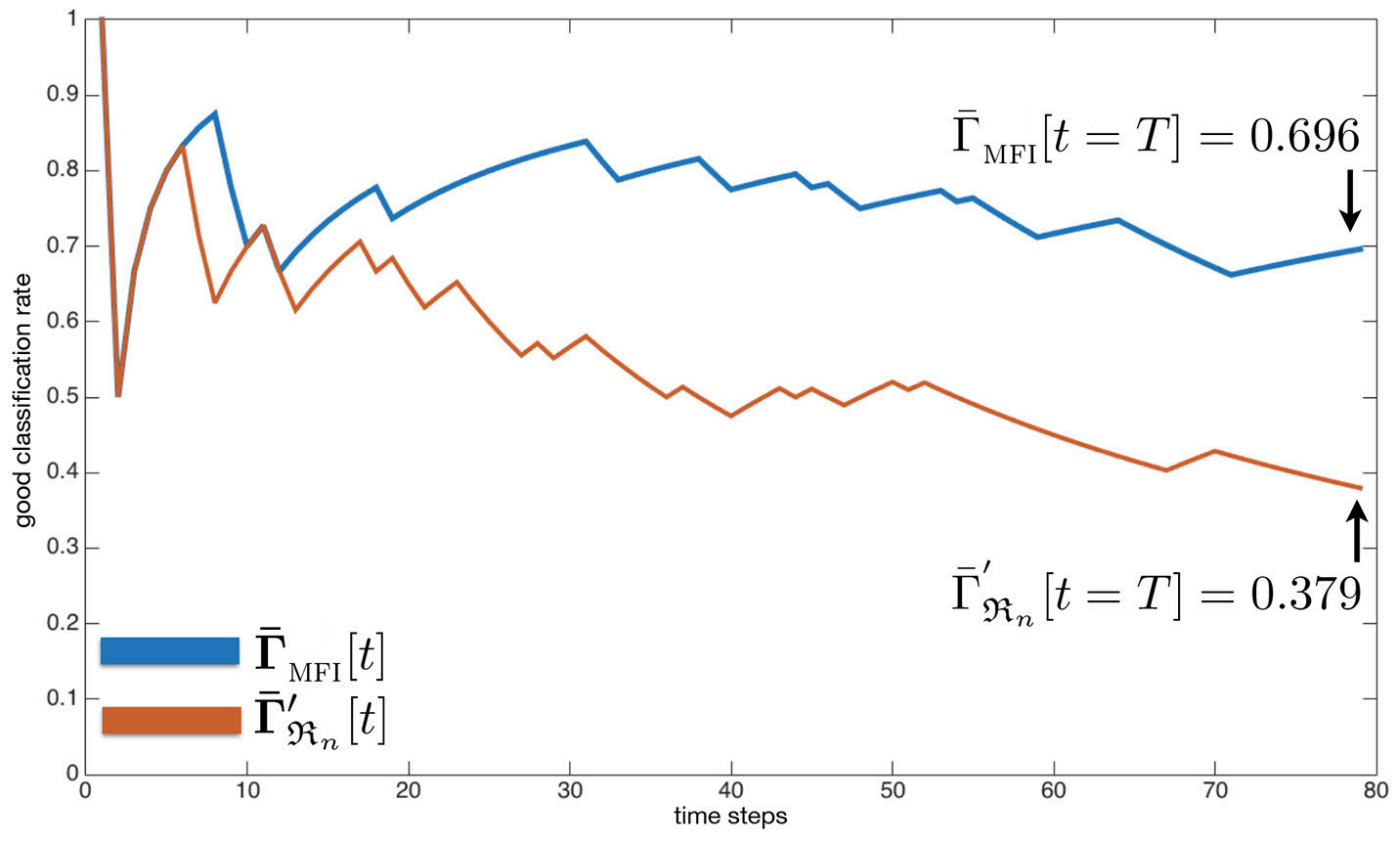
Results of the audiovisual classification (including the inference by the mfi module) obtained by (blue) the HTM system, (red) the naive robot. The two numbers on the right correspond to the value at the end of the exploration.

After having performed numerous evaluation in simulated conditions (see Table 2), this experiment is focused on the evolution of the good audiovisual classification rate along the exploration of a real environment. For that purpose, an environment is set up with five different sources, as presented in Table 3. The three audio classes populating the environment have been selected because of their better experimental recognition rate in the architecture. At each time step, the estimated audiovisual classes provided by the overall HTM system is compared to the ground truth, and for each object. The resulting mean good estimation rate ${\bar{\Gamma }}_{\text{Mfi}}\left[t\right]$, computed over all objects, is plotted against time in Figure 11 (blue line). The same is done for the naive robot, with a mean good estimation rate ${\bar{\Gamma }}_{{ℜ}_{n}}^{\prime }\left[t\right]$ (red line in the same figure). As expected, the proposed HTM system shows the best audiovisual classification rate. Indeed, one can see in Figure 11 that the red line tends to the rate ${\bar{\Gamma }}_{{ℜ}_{n}}^{\prime }=37.9\%$ which is exactly the mean good classification rate of the involved KS. In the same conditions, the mfi module converges to ${\bar{\Gamma }}_{\text{Mfi}}=69.6\%$. In the very beginning of the experiment, both systems exhibit the same performances: the different smoothing involved in the various computations (of the KS outputs, in the motor decisions, etc.) together with silences in the sounds presented to the robot can explain this. But while the naive robot exhibits a constantly decreasing good estimation rate of the audiovisual classes, the mfi module remains relatively robust to the KS classification errors.

**Figure 12:**
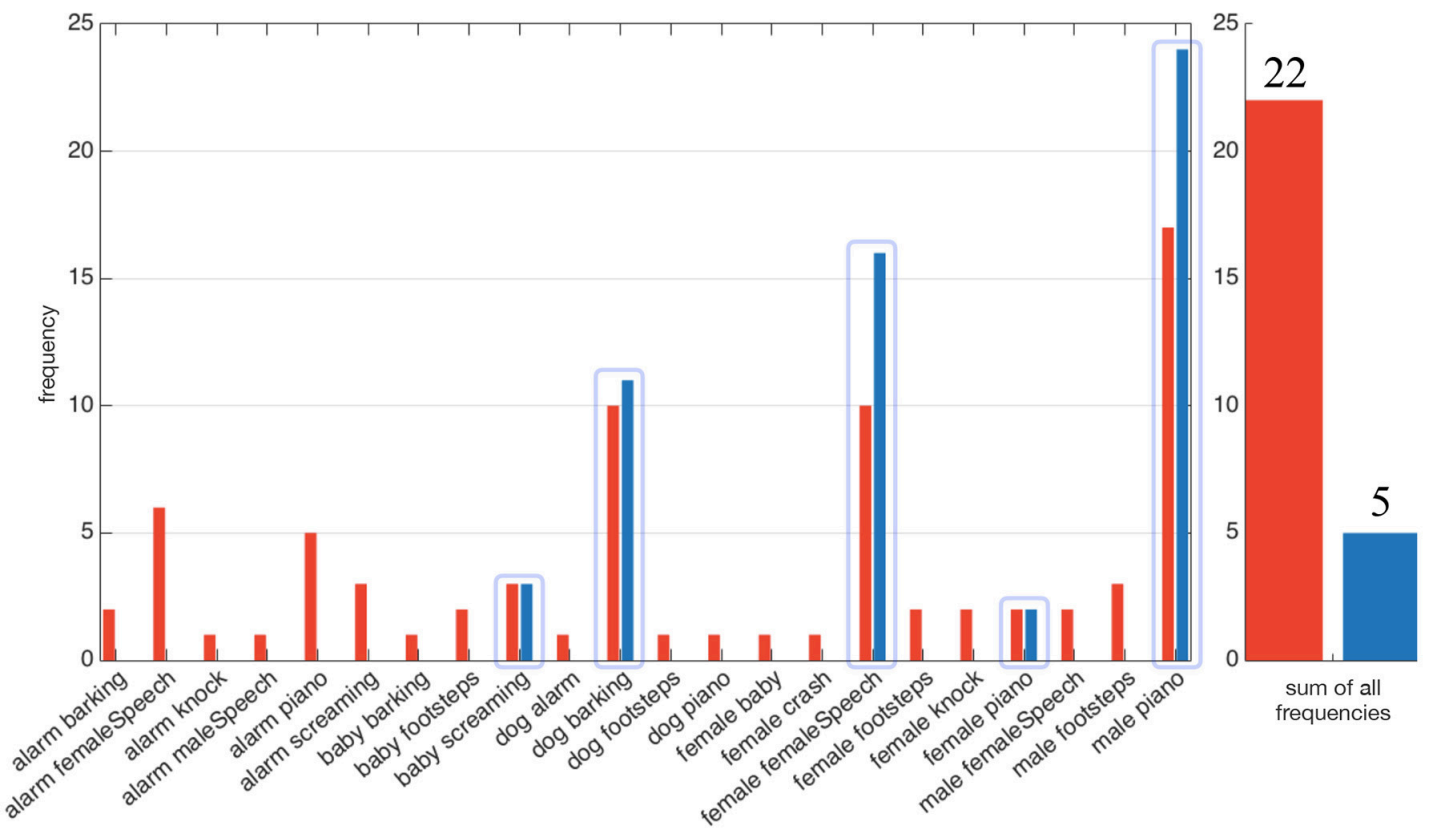
Number and labels of the audiovisual classes created by (blue) the mfi module, and (red) the naive robot. **(Left)** Number of temporal frames (height of bars) during which the audiovisual classes have been categorized. (Light blue rectangles:) Audiovisual classes the two systems have in common. **(Right)** Total number of different audiovisual classes created.

A direct consequence of these good performances of the HTM system can be observed in Figure 12 which plots an histogram of all the audiovisual classes created by both systems (expressed in terms of number of frames). The HTM system is able to considerably narrow the possible audiovisual classes existing in the environment: from 22 by the naive robot, the HTM system narrows it down to only 5. However, one of the class created is erroneous: piano, female has been mistaken with piano, male, but only for a short period of time (two frames, i.e., 1 s). This point has already been discussed in section 3.3.4.

## 5. Conclusion

In this paper, a new system for the modulation of the exploratory behavior of a robot has been proposed. Based on the new notion of Congruence, it takes control of the head movements of a platform to put the robot attention toward audiovisual sources of interest. Additionally, it provides a robust description of the unknown environments explored all along the robot's life and following an unsupervised paradigm. This enriched representation consists, first, in the analysis of audiovisual objects through their relationship to the environments they are perceived in, and secondly, in how much the knowledge the system has about their actual audiovisual class is reliable and robust. Even in the case of classification errors by the audio or visual classifiers in the overall architecture, the system is then able to correctly infer the events' audiovisual classes by actively learning the interlink between the two modalities. All of this is achieved by the two constitutive modules of the HTM, namely the *Dynamic Weighting* module, and the *Multimodal Fusion & Inference* module. Each of them is able to trigger head movements that are used as an attentional reaction and as an active reaction to the need for additional data, respectively. Importantly, the extensive use of head movements is not limited to the sole benefit of the HTM system: audio localization algorithms such as (Nakashima and Mukai, 2005; Hornstein et al., 2006; Ma et al., 2017) relying also on head movements could be connected to the HTM as a top-down feedback unit, thus taking advantage from its motor commands to improve in parallel audio localization performances. The active self-supervised and online learning paradigm the mfi module relies upon, through the use of the *Multimodal Self- Organizing Map*, quickly provides the dw module with robust data while also offering inference abilities whenever a modality is missing (occlusion of the object, for instance). Whereas existing models provide audio-visual inference (Alameda-Pineda and Horaud, 2015) aiming at binding low-level cues of the audio and visual data streams, the mfi module relies only on a higher level of representation of data, a representation that could be used as a top-down feedback to potentially enhance low-level audiovisual fusion algorithm. Additionally, the choice of learning the cross-modal relationship between auditory and visual data in an exclusively unsupervised way can be debated as not being powerful enough (Senocak et al., 2018). However, the results obtained here show significant improvements in the quality of the audiovisual data provided to the dw module without any inclusion of human knowledge. The system performances have been evaluated in realistic simulated conditions, but also on a real robot endowed with binaural audition and vision capabilities. Importantly, the overall architecture of the system, i.e., the Two!Ears software, is made available online as an open source software^2^. The same applies for the proposed HTM system, entirely included inside this architecture^3^.

One of the main limitation of the current implementation is related to its high dependency to the localization experts. Indeed, the overall motor reactions are currently guided by each object azimuth localization, which have been shown precise enough to provide relevant results. Hopefully, binaural sound localization is a research topic by itself, and recent developments in the field show very robust algorithms, even in challenging acoustical conditions. Nevertheless, the robustness to localization errors could be enhanced by using tracking experts able to consolidate the sources position along time. For now, the HTM system is still being developed with the following improvements in mind. First, the definition of an object is currently limited to its audio and visual labels, while it could be enriched with additional information possibly coming from other modalities (emotions, audio pitch, forms and textures, etc.). Importantly, the proposed M-SOM has been designed to easily incorporate such additional parameters in the object definition: a subnetwork can be added for each of them together with their respective weights vectors. Concerning the Dynamic Weighting module, a significant improvement can be made by including the computation of a temporal habituation in order for the robot to not to be stuck in a deadlock kind of situation, as in Figure 8 where, if the scenario goes on forever, the robot would be keeping turning its head toward the crying, male. Finally, the coupling of the HTM system with other cognitive experts in the current framework is still under investigation. So far, the current version of the Two!Ears software does not include others high- level cognitive experts. Nevertheless, the entire HTM system has been conceived with the idea that the motor exploration can also be guided by cognitive elements other than the ones implemented in the system. For instance, a model as the one recently proposed by Lanillos et al. (2015) on attention driven by social interaction, could easily be linked to the HTM, both benefiting from each other: one congruent source could still be focused because of its social interest, whereas a socially non-interesting object could still be focused for its high incongruence.

## Author contributions

All authors listed have made a substantial, direct and intellectual contribution to the work, and approved it for publication.

### Conflict of interest statement

The authors declare that the research was conducted in the absence of any commercial or financial relationships that could be construed as a potential conflict of interest.

## Footnotes

^1^http://www.twoears.eu

^2^https://github.com/TWOEARS

^3^https://github.com/TWOEARS/audio-visual-integration
